# A Uterus‐Inspired Niche Drives Blastocyst Development to the Early Organogenesis

**DOI:** 10.1002/advs.202202282

**Published:** 2022-07-17

**Authors:** Zhen Gu, Jia Guo, Jinglei Zhai, Guihai Feng, Xianning Wang, Zili Gao, Kai Li, Shen Ji, Leyun Wang, Yanhong Xu, Xi Chen, Yiming Wang, Shanshan Guo, Man Yang, Linlin Li, Hua Han, Liyuan Jiang, Yongqiang Wen, Liu Wang, Jie Hao, Wei Li, Shutao Wang, Hongmei Wang, Qi Gu

**Affiliations:** ^1^ State Key Laboratory of Membrane Biology The State Key Laboratory of Stem Cell and Reproductive Biology Institute of Zoology Chinese Academy of Sciences Beijing 100101 P. R. China; ^2^ Department of Chemistry and Biological Engineering University of Science and Technology Beijing Beijing 100083 P. R. China; ^3^ CAS Key Laboratory of Bio‐Inspired Materials and Interfacial Science Technical Institute of Physics and Chemistry Chinese Academy of Sciences Beijing 100190 P. R. China; ^4^ Beijing Institute for Stem Cell and Regenerative Medicine Beijing 100101 P. R. China; ^5^ University of Chinese Academy of Sciences Beijing 100049 P. R. China; ^6^ National Laboratory of Pattern Recognition Institute of Automation Chinese Academy of Sciences Beijing 100190 P. R. China; ^7^ School of Life Sciences Northeast Agricultural University Harbin 150030 P. R. China

**Keywords:** early organogenesis, implantation, in vitro culture (IVC), uterus‐inspired

## Abstract

The fundamental physical features such as the mechanical properties and microstructures of the uterus need to be considered when building in vitro culture platforms to mimic the uterus for embryo implantation and further development but have long been neglected. Here, a uterus‐inspired niche (UN) constructed by grafting collagen gels onto polydimethylsiloxane based on a systematic investigation of a series of parameters (varying concentrations and thicknesses of collagen gel) is established to intrinsically specify and simulate the mechanics and microstructures of the mouse uterus. This brand‐new and unique system is robust in supporting embryo invasion, as evidenced by the special interaction between the embryos and the UN system and successfully promoting E3.5 embryo development into the early organogenesis stage. This platform serves as a powerful tool for developmental biology and tissue engineering.

## Graphical abstract

A uterus‐inspired niche drives blastocyst development to early organogenesis. The uterine horn's modulus and the endometrium microstructure is mimicked to construct a uterus‐inspired niche to support the embryo development to the early organogenesis stage in vitro and to study the invasion of the embryo into collagen fibers for in‐depth studies of embryonic implantation in the future.

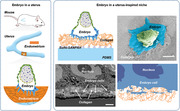

## 1 Introduction

The natural uterine architecture is a highly stratified structure composed of three layers. The top layer of the endometrium, surrounded by the myometrium and perimetrium layers, plays a crucial role in embryo attachment and invasion during implantation.^[^ ^1^ ^]^ Trophectoderm (TE) cells of the blastocyst make the first contact with the uterine epithelium for implantation. For mice, the blastocyst attaches onto the maternal endometrium at embryonic day (E) 4.0‐E4.5 with the mural TE (the part of TE at the opposite sides of the inner cell mass‐ICM), which develops into trophoblast giant cells (TGC) that secrete progesterone/estrogen and matrix metalloproteinases to remodel the extracellular matrix (ECM) of the endometrium and guarantee the successful implantation.^[^ ^2^ ^]^ The polar TE, which is contiguous with ICM, will develop into the extraembryonic ectoderm (ExE) and eventually the placenta, while the ICM gives rise to the epiblast and hypoblast, which will further contribute to the main embryonic body and yolk sac, respectively.^[^ ^3^, ^4^, ^5^ ^]^

Embryo implantation is the prerequisite for proper embryo development: the formation of EPI cavity and ExE cavity, the elongation of “egg cylinder” structure (with proamniotic cavity formation), gastrulation, neurulation, and the heart tube formation, etc.^[^ ^6^, ^7^ ^]^ Errors in any of these events may lead to abnormalities in the embryo and even pregnancy failure.^[^ ^8^ ^]^ To visualize and manipulate the progress of implantation and the postimplantation embryonic development, the technique of in vitro culture (IVC) of embryos has been proposed since the 1930s.^[^ ^9^, ^10^, ^11^, ^12^, ^13^, ^14^, ^15^, ^16^, ^17^, ^18^, ^19^, ^20^, ^21^, ^22^, ^23^ ^]^ Various substrates have been tried to support preimplantation and postimplantation mouse embryos development in vitro, such as uterine stromal cells, biomaterials, and integrating these two.^[^ ^11^, ^12^, ^13^, ^14^, ^15^, ^16^, ^18^, ^23^, ^24^, ^25^, ^26^, ^27^, ^28^, ^29^ ^]^ The matrix proteins used to coat Petri dishes include agar, collagen, laminin, fibronectin, and Matrigel matrix.^[^ ^23^, ^24^, ^25^, ^26^, ^27^, ^28^ ^]^ Currently, E3.5 embryos can be cultured in vitro up to E6.75 by Zernicka‐Goetz.^[^ ^18^, ^25^ ^]^ While E4.5 embryo development supported by Matrigel‐collagen mixture^[^ ^14^ ^]^ or DexMA or PEG^[^ ^15^ ^]^ can be cultured in vitro up to E6.0 by Takashi Hiiragi^[^ ^14^ ^]^ and Aguilera‐Castrejon pushed E5.5 embryos to E11.0 in vitro using a roller culture platform.^[^ ^16^ ^]^ However, it is difficult for the in vitro embryo development for a long time from the preimplantation stage, and few studies have found that the in vitro culture system can both complete the early trophoblast cell invasion into the matrix and support E3.5 embryo development beyond the postgastrulation stage.

The mechanical properties and microstructures of the uterus determine the outcome of implantation. However, few studies have focused on mimicking the uterine environment based on these characteristics.^[^ ^17^, ^18^ ^]^ The Young's modulus of the oviduct and uterine epithelium is ≈100–1000 Pa, while a conventional polystyrene culture plate is around 1 GPa,^[^ ^30^, ^31^ ^]^ which suggests that the culture plate is not suitable for the in vitro culture of embryos. Finding proper combinations of substrates to mimic the uterine mechanical properties and microstructures closely will provide the possible platform to support embryo in vitro development. Collagen can form a porous fibrous structure to help cells attach, develop and migrate, and can be widely used in the construction of biomimetic environments.^[^ ^32^, ^33^ ^]^ Polydimethylsiloxane (PDMS) is commonly used in cell culture because of its stability, transparency, flexibility, and biocompatibility.^[^ ^32^ ^]^ Although PDMS^[^ ^34^ ^]^ and collagen^[^ ^14^ ^]^ have been used for in vitro culture, it is still a challenge to construct a uterus‐inspired microenvironment from the mechanics and microstructures' perspective to achieve longer‐term in vitro development of embryos.

**Figure 1:**
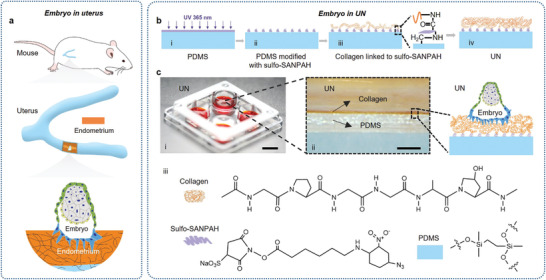
Design of a UN system based on the in vivo uterine microenvironments. a) Schematic diagram of embryo adhering to the endometrium of the uterus. Forest green, trophectoderm; White smoke, epiblast; Lemon, primitive endoderm cells; Electric blue, trophectoderm‐derived cells; Tiger, the endometrium. b) Schematic showing the preparation procedure of UN systems. c) Schematic illustration of the UN for embryo culture in vitro. i) digital image of 4‐well petri dish containing the UN system. Scale bar, 1 cm. ii) a magnified view of the cross section of the culture substrate with a schematic of the embryo attachment site. Scale bar, 500 µm. iii) molecular structures of collagen, PDMS and Sulfo‐SANPAH as the covalent binding links between collagen and PDMS for UN systems.

In this study, a uterus‐inspired niche, which closely mimics the E3.5 uterus in terms of physical modulus and microstructures, was constructed by placing a layer of collagen on PDMS (**Figure** **1**). Using this robust and unique system, the invasion of the embryo into the matrix can be well observed, and the embryos displayed high developmental efficiency in a series of critical developmental events and developed into the stage of early organogenesis.

## 2 Results and Discussion

### 2.1 Design of UN Systems

**Figure 2:**
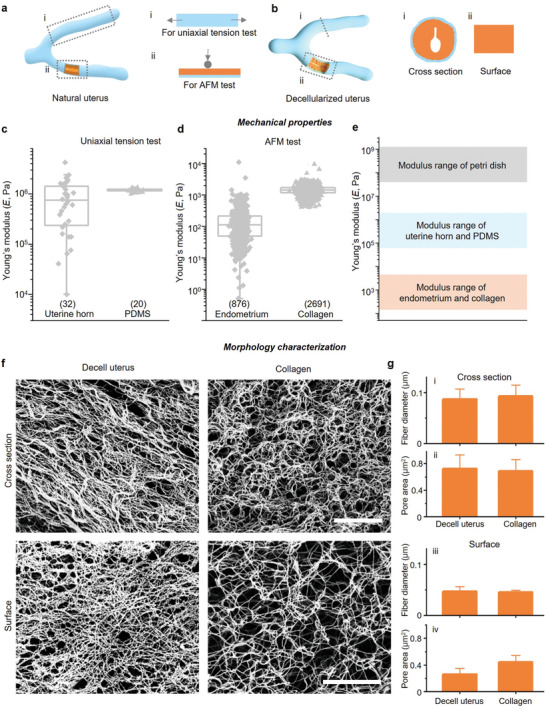
Mechanical properties testing and morphological characterizations of the UN system. a) Schematic diagram of evaluating mechanical properties in two modes including tension and indentation by uniaxial tension and AFM tests. Tiger, endometrium; Maya, natural uterus; Baby blue, decell uterus. b) Schematic diagram of the cross sections and surface of the decell uterus. c) Measurement of Young's modulus (*E*) of the E3.5 uterine horn (*n* = 32) and PDMS (*n* = 20, the mass ratio of the matrix and crosslinker was 10:1) by uniaxial tension test. d) Measurement of Young's modulus (*E*) of endometrium (E3.5) (*n* = 6) and collagen (*n* = 6, 7.5 mg mL^−1^, 46 ± 10 µm in thickness) in by AFM test. e) Range of Young's modulus of the uterine horn, endometrium, PDMS, collagen, and Petri dish. f) SEM images of the cross section and surface of decell E3.5 uterus matrix and collagen. Scale bars, 3 µm (upper panel) and 5 µm (lower panel). g) The diameters of fibers (i and iii) and pore areas of the fibrous mesh (ii and iv) for SEM images of decell E3.5 uterus matrix and collagen with the cross section and surface, respectively. ImageJ was used for analyzing the diameters of fibers and pore areas of samples. For f) and g), decell E3.5 uterus matrix: *n* = 4 and 3 from uterus samples for cross section and surface, respectively, 5 images of each sample are counted; collagen: *n* = 3 from collagen gels for cross section and surface, respectively, 5 images of each sample are counted, 7.5 mg mL^−1^, 46 ± 10 µm in thickness.

Embryo implantation and embryo development are highly dependent on the microenvironment, such as the mechanical properties (**Figure** **2**a) and structural features of the uterus (Figure 2b). To elucidate the mechanical properties of the uterus during peri‐implantation, we collected the uterine tissues from adult female mice at day 3.5 post coitum (E3.5) and measured the moduli of the entire uterine horn and the endometrium surface (Figure 2a; and Figure S1, Supporting Information) by the uniaxial tension test on a universal testing machine (Figure S2a and Movie S1, Supporting Information) and the atomic force microscopy (AFM) test (Figure S2b, Supporting Information). We found (Figure S2, Supporting Information) that the elastic modulus (Young's modulus) ranges of the uterine horn and the uterine endometrium were megapascal (MPa) and kilopascal (kPa) order of magnitudes, respectively (Figure 2c–e). To reproduce the physical properties of the endometrium and the uterine horn, we loaded Collagen I liquid onto PDMS for gelation (Figure 1a,b). Collagen and PDMS were covalently bonded to establish the uterus‐inspired niche system (Figure 1c). We modulated the key parameters of the upper‐layer collagen hydrogel, namely, the thickness and the concentration, to develop a series of UN systems, including PDCO‐C, PDCO, PDCO‐T, the UN with different thicknesses of collagen (PDCO‐C: PDMS (496 ± 156 µm thick) coated with collagen. PDCO: bottom layer PDMS (496 ± 156 µm thick) bonded with the top layer collagen (type I, 7.5 mg mL^−1^, 46 ± 10 µm thick). PDCO‐T: bottom layer PDMS (496 ± 156 µm thick) bonded with the top layer collagen (7.5 mg mL^−1^, 417 ± 137 µm thick)) and PDCO‐L, PDCO, PDCO‐H, the UN with varying concentrations 5, 7.5, and 10 mg mL^−1^, respectively, of collagen (Figure S3, Supporting Information). PDCO‐L, PDCO, and PDCO‐H are fabricated with top layer collagen gel (46 ± 10 µm thick) at the concentrations of 5.0, 7.5, and 10.0 mg mL^−1^, respectively on bottom layer PDMS (496 ± 156 µm thick). PDMS (PD) with a mixing ratio of 10:1 (base to curing agent by mass) was selected as the bottom because its elastic modulus was at MPa level, which is similar to that of the uterine horn (Figure 2c,e). The density of the collagen polymer network could be adjusted to modulate the elastic modulus of the endometrium. In order to optimize a proper concentration of collagen, we measured the elastic modulus of collagen at 5, 7.5, and 10 mg mL^−1^ and found that 7.5 mg mL^−1^ collagen exhibited a similar elastic modulus as the endometrium based on AFM (Figure 2e; and Figures S2 and S4, Supporting Information).

To better mimic the uterine architecture, we carefully studied the microstructure of the decell uterus by scanning electron microscopy (SEM) and field emission scanning electron microscopy (FE‐SEM) (Figure 2b). The diameter of the fibers (*D*) and the size of the pore cross sections (*S*) of the fibrous mesh of the decellularized uterus (decell uterus) were 0.09 ± 0.02 µm and 0.72 ± 0.21 µm^2^, respectively (Figure 2f,g; and Figure S5, and Movie S2, Supporting Information). The SEM data of lumen surface and cross section of the uterus proved the retention of delicate ECM structures and tissue integrity (Figure S6 and Movies S3 and S4, Supporting Information). Then, we tested different UN systems and found that the collagen at a concentration of 7.5 mg mL^−1^ and a thickness of 46 ± 10 µm precisely resembled the uterine matrix in terms of the fiber diameter and pore area in the surface and cross sections (*D* = 0.09 ± 0.02 µm and *S* = 0.69 ± 0.17 µm^2^) (Figure 2g; and Figure S7, Supporting Information). Based on the above results, we chose the UN system PDCO, a layer of collagen (7.5 mg mL^−1^, 46 ± 10 µm thick) grafting onto the PDMS (496 ± 156 µm thick) to mimic the modulus of the uterus horn and endometrium and microstructure of the endometrium during pregnancy.

### 2.2 Comparison of Embryonic Development in Different UN Systems

To investigate whether the UN system PDCO is genuinely superior in supporting embryo development, we cultured mouse E3.5 embryos on PDCO and the traditional Petri dish, PD or CO (Figure S8, Supporting Information). Compared with embryos cultured on Petri dish (16 ± 10%), PD (16 ± 8%), and CO (13 ± 6%), a higher portion of the embryos grown on PDCO (36 ± 6%) formed two cavities (EPI cavity and ExE cavity) on IVC day 3 (Figure S8b,c, Supporting Information). Additionally, a higher ratio of embryos cultured on PDCO formed egg cylinders (47 ± 15% for PDCO compared with 32 ± 5% for Petri dish, 32 ± 11% for PD, and 3 ± 2% for CO) or developed to the heartbeat‐like stage (11 ± 5% for PDCO and 1 ± 1% for Petri dish, 0% for PD and 1 ± 2% for CO) as compared with those on Petri dish, PD or CO (Figure S8c, Supporting Information).

To further determine whether PDCO is more robust than the other UN systems with different concentrations and thicknesses of collagen in supporting embryo development, we cultured E3.5 embryos on PDCO‐L, PDCO, and PDCO‐H, as well as PDCO‐C, PDCO, and PDCO‐T over 8 days (Figure S9a, Supporting Information). We did not observe a noticeable difference in the attachment efficiency of the embryos in different systems. However, compared with PDCO‐L or PDCO‐H, embryos cultured on PDCO showed higher rates of the formations of two cavities on IVC day 3 and egg cylinder structures on IVC day 5 (Figure S9b,c, Supporting Information). Furthermore, compared with PDCO‐C or PDCO‐T, more embryos on PDCO formed two cavities on IVC day 3, which led to an increased number of embryos with egg cylinder on IVC day 5 (Figure S9b,d, Supporting Information). The above results suggested the impact of the concentration and thickness of collagen in the UN system on in vitro embryonic development and illustrated further that PDCO with collagen at a concentration of 7.5 mg mL^−1^ and a thickness of 46 ± 10 µm (Figure 2g; and Figure S7, Supporting Information), was more effective for supporting embryonic growth. Altogether, we confirmed the successful establishment of an in vitro embryo culture system (UN), which closely mimics the moduli of the uterus horn and endometrium and the microstructure of the endometrium.

### 2.3 Morphology and Transcriptome Features of In Vitro Embryos on PDCO

**Figure 3:**
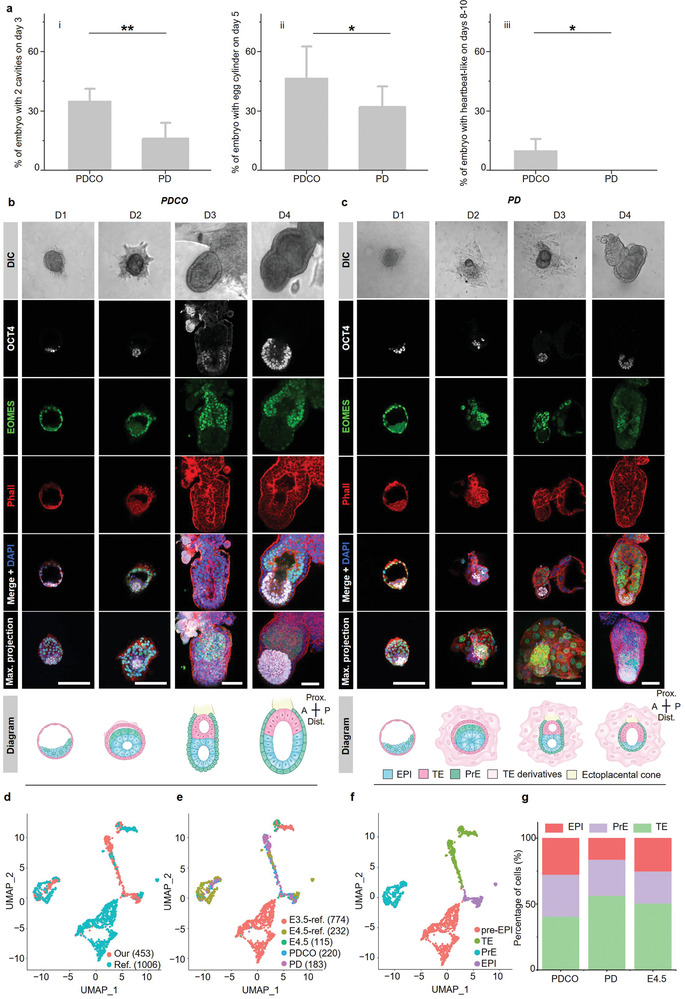
PDCO system is conducive to embryonic development. a) Comparison of embryo development on PDCO and PD systems. The percentages of embryos with 2 cavities (EPI cavity and ExE cavity) on IVC day 3 i), egg cylinder on IVC day 5 ii), and heartbeat‐like beating on IVC days 8–10 (iii) were statistically analyzed between PDCO (*n* = 71 embryos) and PD (*n* = 64 embryos). Two‐tailed Student's *t*‐test. *P < 0.05, **P < 0.01. b,c) Representative images of embryonic morphology from IVC day 1 (D1) to day 4 (D4) on PDCO (*n* = 15) compared to PD (*n* = 15). Top panel, differential interference contrast (DIC) images showing time‐course of embryo development on PDCO and PD. Middle panels, the representative confocal Z section images, and a maximum projection of the embryos were stained by EPI marker OCT4 (white), trophoblast marker EOMES (green), and cytoskeleton marker Phall (red) with DAPI (blue) to visualize nuclei. Scale bars = 100 µm. Bottom panel, schematic illustration of morphological changes during D1–D4. D, in vitro culture day. d) UMAP embedding overlay showing the distribution of single cells from our database (Our) and referenced data resources.^[^ ^38^ ^]^ e) UMAP embedding overlay showing the 518 single cells from PDCO and PD embryos on IVC day 2 and in vivo E4.5 embryos, and referenced 1006 cells from E3.4 and E4.5 embryos.^[^ ^38^ ^]^ E, embryonic day. f) UMAP showing the four major clusters of embryo cells identified by different gene expressions. EPI, epiblast; PrE, primitive endoderm; TE, trophectoderm. pre‐EPI, epiblast at preimplantation stage. g) Percentages of EPI, PrE, and TE cells to total cells in the embryos cultured on PDCO or PD and E4.5 embryos.

To further confirm and specify the superiority of the PDCO system for embryonic development (**Figure** **3**a), we cultured E3.5 embryos on both the PDCO and PD for 1–4 days. The embryos were collected and immunostained with antibodies against OCT4 (EPI and derivatives), EOMES (trophoblast cells), and phalloidin (cytoskeleton) (Figure 3b,c). On IVC day 1, the epiblast (OCT4 positive) of the embryos cultured on both PDCO and PD formed a tight core at the center of the embryo, which was surrounded by EOMES positive trophoblasts. The blastocoel was also clearly identifiable in both PDCO and PD groups (Figure 3b, 1st column (from left to right); Figure 3c, 1st column (from left to right)). On IVC day 2, the embryos cultured on PDCO (PDCO embryos) increased in size and formed a cavity at the center of the OCT4 expressed epiblast, namely, the EPI cavity. The epiblast formed a rosette‐like structure similar to that in normal E4.5 embryos.^[^ ^25^ ^]^ Trophoblasts of PDCO embryos with continuous EOMES expression organized themselves to surround the epiblast and blastocoel (Figure 3b, 2nd column (from left to right)). Compared with PDCO embryos, the trophoblasts of the embryos on PD adopted a flattened structure with large nuclei. The blastocoel collapsed, and the epiblast labeled by OCT4 formed a disorganized sphere without an identifiable rosette‐like shape (Figure 3c, 2nd column (from left to right)).

On IVC day 3, PDCO embryos elongated along the proximal distal axis, and their features were similar to the structure of normal in vivo embryos at E5.5.^[^ ^34^ ^]^ The EPI cavity expanded at the center of the epiblast, and the ExE cavity emerged at the center of the EOMES positive trophoblasts (Figure 3b, 3rd column (from left to right)). Embryos on PD showed trophoblasts extended from the embryo and spread on the substrate. In addition, the OCT4 positive cells in PD embryos showed limited proliferation, the growth of the putative egg cylinder structure was restricted, and the ExE cavity or EPI cavity was too vague to be distinguished (Figure 3c, 3rd column (from left to right)). On IVC day 4, the ExE cavity and EPI cavity merged to form proamniotic cavities in PDCO embryos similar to in vivo E6.0 embryos^[^ ^35^ ^]^ (Figure 3b, last column (from left to right)). Compared with PDCO embryos, the proamniotic cavity in PD embryos on IVC day 4 was less pronounced (Figure 3c, last column (from left to right)). The unified development of embryonic and extraembryonic tissues is vital for early embryogenesis.^[^ ^36^, ^37^ ^]^ The differentiation imbalance between embryonic and extraembryonic tissues on PD might lead to the degeneration of the epiblast much disorganization to develop to a more advanced stage.

To further identify the transcriptome features of embryos cultured on PDCO or PD, we collected 518 single cells from PDCO and PD embryos on IVC day 2 and in vivo E4.5 embryos and performed single‐cell RNA‐seq (scRNA‐seq) analyses (Figure 3d–g). We compared the transcriptional signatures of these single cells with those from reported in vivo E3.5 and E4.5 counterparts (1006 cells)^[^ ^38^ ^]^ and applied uniform manifold approximation and projection (UMAP) dimension reduction analysis on the combined datasets using the Seurat package to visualize groups of transcriptomically similar cells. Based on the specific marker genes of the cells, UMAP revealed that cells from IVC and in vivo embryos were clustered into 4 primary cell types, the pre‐implantation epiblast and epiblast (pre‐EPI and EPI, *Pou5f1, Nanog, Klf2*, and *Esrrb*), the trophectoderm (TE, *Cdx2, Eomes, Elf5, Dmkn, Gata3*, and *Tspan8*), and the primitive endoderm (PrE, *Sox7, Foxq1, Lhx1, Gata4, Gata6*, and *Runx1*) (Figure 3f; and Figure S10, Supporting Information). Although no significant differences in cell types were found between embryos cultured on PD and PDCO, the percentages of epiblast, primitive endoderm, and trophoblast cells in PDCO embryos were more consistent with those in in vivo E4.5 embryos (Figure 3g). However, embryos cultured on PD contained more than 50% trophoblast cells and less than 20% epiblast cells (Figure 3g). The imbalanced growth of embryonic and extraembryonic tissues in PD embryos might impede their advanced embryonic development.

### 2.4 Interactions Between Mouse Embryos and UN During Peri‐Implantation Stage

**Figure 4:**
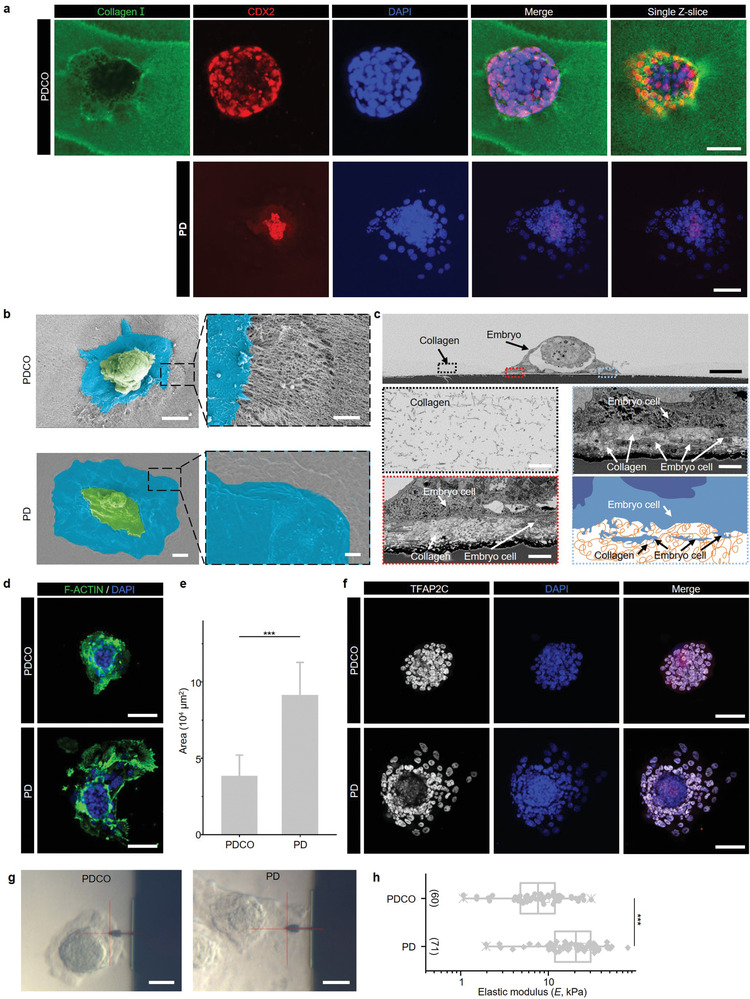
Embryo invasion into PDCO or PD and trophoblast status on IVC day 2. a) Top view of the embryos invading into PDCO (*n* = 10) or PD (*n* = 10) on IVC day 2 by immunostaining using antibodies of CDX2 (red, labeling trophoblast) and Collagen I (green, labeling the top layer of PDCO). Scale bars, 100 µm. b) SEM images of the interfaces between embryos and the PDCO (*n* = 5) or PD (*n* = 5) substrates. The embryo‐ and TE‐derived cells are colored green and dark blue, respectively. Scale bars, 50 µm (left panel) and 10 µm (right panel). c) Sectional SEM imaging the cross section of embryo invading into collagen gels of the PDCO. The detailed zooming information of collagen and embryo cells is indicated in the gray dotted box and red dotted box, respectively, which are also summarized by the Air force dotted line box schematic. White smoke, collagen; Iron: embryo cells. Scale bars, 50 µm (top panel) and 3 µm (bottom panel). d) Immunostaining of F‐actin (green) for PDCO and PD embryos. Scale bars, 100 µm. e) Statistics of the embryonic spreading area of PDCO (*n* = 12) and PD (*n* = 12) embryos on IVC day 2, respectively. Two‐tailed Student's *t*‐test. ***P < 0.001. f) Immunostaining of embryos on PDCO (*n* = 12) and PD (*n* = 12) using antibodies of TFAP2C (gray, labeling TE) and DAPI (blue, labeling the nuclei). Scale bars, 100 µm. g) DIC images of embryos on PDCO or PD during AFM test. Scale bars, 50 µm. h) The statistic analyses of elastic modulus of the trophoblasts from embryos on PDCO or PD. *n* = 5 embryos for each condition. Two‐tailed Student's *t*‐test.***P < 0.001.

The advantages of PDCO for embryo support were pronounced when trophoblasts attached to the PDCO substrate and displayed a more organized morphology than the PD trophoblasts. The staining results of Collagen I and CDX2 demonstrated that the attached embryos on PDCO showed a dome‐shaped morphology attached to and invaded into the PDCO substrate, forming a curved region (or an identifiable pit), where TE cells and collagen fibers interacted with each other (**Figure** **4**a, upper panel; and Figure S11a–d and Movie S5, Supporting Information). However, the embryos on PD only spread on the surface with prominent nuclei and a significantly lower proportion of CDX2 positive cells, while without obvious invasion signs or pit (Figure 4a, bottom panel; and Figure S11e and Movie S6, Supporting Information).

SEM and sectional SEM imaging were performed to further assess the physical connection between embryos and materials during embryo attachment and invasion. The overall images of SEM indicated that some trophoblasts of PDCO embryos expanded out of the embryo and formed a radial microvilli‐like structure which interacted with collagen fibers. However, PD embryos attached to the surface of the PD exhibited a flat morphology and smooth expanding outer edges (Figure 4b). Furthermore, the sectional SEM results showed that the flexural and amorphous cellular membrane of trophoblasts in PDCO embryos mixed and integrated with collagen, which could be distinctively identified by its boundary along the fibers, offering the excellent invasion of trophoblasts into collagen (Figure 4c; and Figure S12, Supporting Information). However, the embryos on PD labeled by Phall displayed a relatively larger spreading area with flag‐like morphology (Figure 4d,e), and immunofluorescent staining confirmed that the spreading area was dominated by TGCs (TFAP2C positive, Figure 4f).^[^ ^39^, ^40^ ^]^ Based on AFM measurements, we found that the trophoblasts on PDCO exhibited a lower elastic modulus than the trophoblasts on PD (Figure 4g,h), which indicated that cell mechanical properties can be affected by the environment and contributed to the invasion.^[^ ^41^, ^42^, ^43^ ^]^ We then performed scRNA‐seq to illustrate how early the difference in gene signature occurred, which leads to the differences in the developmental potency between PDCO and PD embryos. In Figure S13a,b, Supporting Information, we found the expression of pregnancy‐related hormone genes, and the implantation‐related genes in TGC, were comparable between E4.5 natural embryos, PDCO embryos, and PD embryos, suggesting that the TGC in PDCO or PD embryos could secrete hormones and display invasion like natural TGCs. There is not increased or decreased trend among E4.5, PDCO, and PD embryos, but the expression of these genes was higher than E3.5 embryos. The expression of these genes might not impact the development potential of PDCO or PD embryos. Still, there are differences of the genes related to “oxidation‐reduction process,” “cell proliferation,” “negative regulation of apoptotic” (Figure S13c, Supporting Information). However, analysis of the differentially expressed genes of embryos on PD and PDCO revealed that the genes upregulated in PDCO embryos were enriched in categories, including negative regulation of the intrinsic apoptotic signaling pathway, the oxidation‐reduction process, cell proliferation, cell migration, embryonic development in utero, etc. (Figure S14, Supporting Information).

### 2.5 Embryonic Gastrulation and Early Organogenesis Supported by PDCO

**Figure 5:**
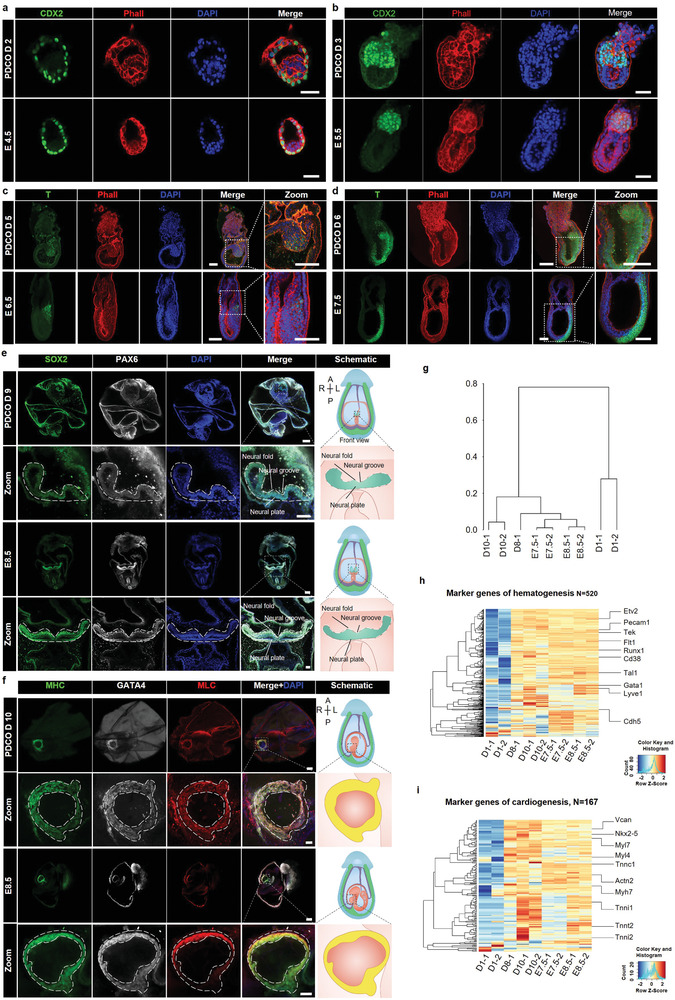
Immunostaining and gene expression of IVC embryos on PDCO from the postimplantation to early organogenesis stage. a,b) IVC day 2 and day 3 embryos on PDCO and in vivo E4.5 and E5.5 embryos labeled by antibodies of CDX2 (green, labeling trophoblast) and Phall (red, labeling cytoskeleton) with nucleus visualization by DAPI (blue). *n* = 15 embryos per condition. Scale bars, 50 µm. c,d) IVC day 5 and day 6 embryos on PDCO and in vivo E6.5 and E7.5 embryos are stained by antibodies of T (green, labeling primitive streak cells), and Phall (red). Dotted line boxes, magnified region. *n* = 20 embryos per condition. Scale bars, 100 µm. e) Left panel: PDCO embryos on IVC day 9 and in vivo E8.5 embryos are stained by antibodies of SOX2 (green, labeling the neural ectoderm cells) and PAX6 (gray, labeling the neural tube cells). Higher‐magnification images of the boxed areas are shown at the bottom. The dotted line boxes (zoomed images) indicate the neural structure. Scale bars, 200 µm (main images), 70 µm (zoomed images). n = 20 embryos per condition. Right panel: schematic showing the front views of PDCO embryo on day 9 (upper) and E8.5 embryo (lower) and the corresponding magnified views of the neural structures. f) Left panel: PDCO embryos on IVC day 10 are stained by antibodies of cardiomyocyte specific markers including MHC (green), GATA4 (gray), and MLC (red), compared to in vivo E8.5 embryos. Higher‐magnification images of the boxed areas are shown at the bottom. The dotted line boxes (zoomed images) indicate the heart tube‐like structure. *n* = 20 embryos per condition. Scale bars, 200 µm (main images), 50 µm (zoomed images). Right panel: schematic showing the side views of PDCO embryo on day 10 (upper) and E8.5 embryo (lower) and the corresponding magnified views of the cardiomyocyte structures. g) The dendrogram unsupervised hierarchically clustered of 9 samples using whole genome‐wide genes with more than 1 FPKM in at least one sample. D1‐1,2 for PDCO embryos on IVC day 1; D8‐1 for PDCO embryos on IVC day 8; D10‐1,‐2 for PDCO embryos on IVC day 10; E7.5‐1,‐2 for in vivo embryos at E7.5 and E8.5‐1,‐2 for in vivo embryos at E8.5. D1‐1,‐2 served as the negative control for the embryos at advanced developmental stages. h,i) Clustering analysis of RNA‐seq results. The heatmaps with scaled expression of specific marker genes related to hematogenesis h) and organogenesis i) in embryos at different developmental stages in vivo or in vitro.

In order to characterize the overall morphology and detailed developmental events in embryos cultured on PDCO, the embryos on day 2‐day 10 and the in vivo E4.5‐E8.5 embryos were collected for immunofluorescent staining. Firstly, we stained CDX2, SOX2, and Phalloidin (Phall) antibodies to label the trophoblast, epiblast, and cytoskeleton, respectively of PDCO embryos on IVC day 2 and day 3, as well as E4.5 and E5.5 embryos. The immunofluorescence results suggested that the morphology of IVC day 2 embryos was similar to in vivo E4.5 embryos (**Figure** **5**a; and Figure S15a, Supporting Information), which was consistent with the findings of scRNA‐seq (Figures 3d–g and 5a). The embryos of PDCO on IVC day 3 shared morphological similarity with the in vivo E5.5 embryos, which contained the EPI cavity and ExE cavity as illustrated by immunostaining of CDX2, SOX2, and Phall (Figure 5b; and Figure S15b, Supporting Information). In PDCO embryos of IVC day 5, T‐positive cells appeared at the posterior region of the egg‐cylinder structure and generally migrated toward the anterior region on IVC day 6, suggesting that these embryos developed into the gastrulation stage as E6.5‐E7.5 in vivo embryos (Figure 5c,d; and Figure S15c,d, Supporting Information). We also observed the emergence of structures resembling early organogenesis, specifically, in PDCO embryos of IVC day 9, the early neural‐like structures (SOX2+/PAX6+) such as neural fold, neural groove, and neural plate could be identified by immunofluorescence staining (Figure 5e). Interestingly, we observed the beating of aggregated cardiomyocytes in embryos cultured in vitro (Movie S7, Supporting Information), and we suspect that in vitro embryos may have reached early organogenesis stage. Although there is a gap between the in vitro embryos and E8.5 embryos, for the convenience of expression, we still choose the in vitro and E8.5 embryos for identification and comparison. We immunostained the embryos with antibodies against myosin heavy chain (MHC), myosin light chain (MLC), and GATA binding protein 4 (GATA4)^[^ ^16^ ^]^ and found that the heartbeat‐like region of the embryo contained a cluster of MHC, ML,C and GATA4 triple positive cells arranged in a circular hollow or chamber, akin to the early heart area structure in normal E8.5 embryos (Figure 5f; and Figure S15e, Supporting Information). Furthermore, an expanded yolk sac with distinguished CD31 positive endothelial cells and red blood cells was also observed (Figure S15f,g, Supporting Information). These morphological results suggest that PDCO can support embryos in vitro cultured for 10 days, and the embryos could develop into the early organogenesis stage.

**Figure 6:**
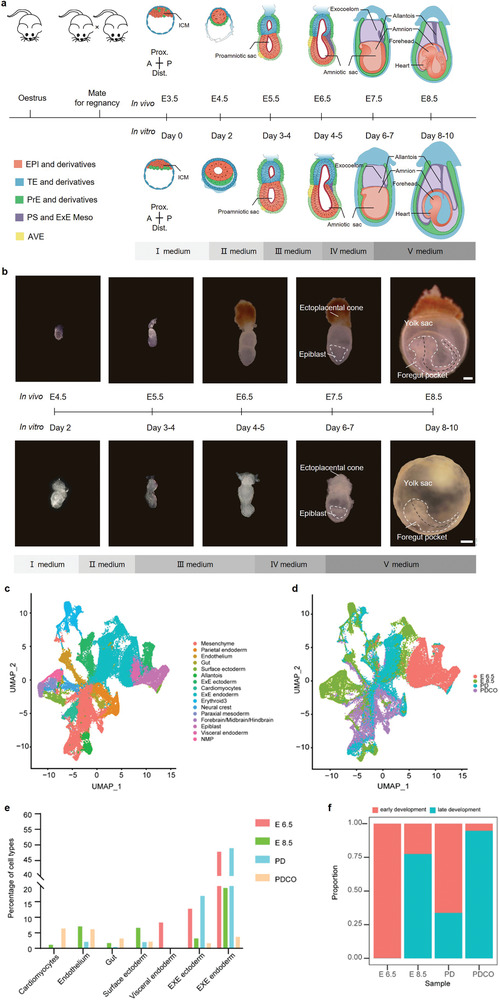
IVC mouse embryos recapitulating the developmental events from peri‐implantation to early cardiogenesis stage. a) Scheme of mouse embryogenesis from peri‐implantation to early organogenesis stage. E, embryonic day. b) Representative bright‐field images of mouse embryos growing at the indicated stages in vivo and in vitro. The white dotted line indicates the embryo bodies. Scale bars = 100 µm. Day, in vitro culture day. The black dotted line indicates the length of the A‐P axis in embryo regions. c) Uniform manifold approximation and projection (UMAP) plot showing the cell atlas. Cells are colored by their cell‐types annotation according to the legend below. E6.5 (14 935 cells), E8.5 (15 116 cells), PDCO (21 546 cells), PD (16 989 cells). d) UMAP embedding overlay showing the origins of cells from indicated embryos. e) Percentages of different cell types among total cells in in vivo and in vitro embryos. f) Percentages of early and late embryonic development cells among total cells in in vivo and in vitro embryos. Early development, cell types in E6.5 embryos, Late development, the other cell types except in E6.5 embryos.

To further confirm this, we collected PDCO embryos on IVC day 8‐day10 together with in vivo E7.5‐E8.5 embryos and performed the bulk RNA sequencing, statistical analyzing and hierarchical clustering. Unsupervised clustering analysis of the overall gene expression patterns showed that in vivo E7.5 and E8.5 embryos first cluster among themselves and then with the PDCO embryos of day 8 and day 10, indicating the similarity between E7.5 and E8.5 embryos on one hand, and the similarity between PDCO embryos of day 8 and day 10 and E7.5 and E8.5 embryos on the other hand (Figure 5g). To visualize the expression of cell type specific markers, gene‐wise clustering was integrated with sample‐wise clustering on the heatmaps. Clear sample clusters with cell type‐specific manners were observed, and there were 520 hematogenesis‐related genes, such as *Etv2, Pecam1, Tek*, and *Flt1* (Figure 5h), and 167 cardiogenesis‐related genes, such as *Vcan, Nkx2‐5, Myl7, Tnnc1*, etc. (Figure 5i) expressed in PDCO embryos of day 8–10 and in vivo E7.5‐E8.5 embryos. In addition, specific marker genes upregulated in gastrulation (*Eomes, T, Ism2, Dhps*, etc., in primitive streak cells), neurulation (*Pax6, Tuba3, Sox21*, etc., in early neural cells; *Sox10, Pax3*, etc., in neural crest), and notochord formation (*Chrd, Noto, T*, etc., in axial mesoderm and notochord cells) could be detected in both the PDCO embryos of day 8–10 and in vivo E7.5‐E8.5 embryos (Figure S16, Supporting Information). Above all, the chronological patterns of cell lineage differentiation and the morphogenesis of PDCO embryos and their in vivo embryos were correspondingly illustrated in **Figure** **6**. The lengths of E8.5 and PDCO (day 8–10) embryos were similar along the anterior–posterior (A–P) axis (Figure S16e, Supporting Information), and the size of PDCO (day 8–10) embryo regions were similar to the E8.5 natural embryos (Figure S16f, Supporting Information). To examine the developmental status and compare the cell component of PDCO day 8–10 embryos, we collected single cells from PDCO day 8–10 embryos, PD day 8–10 embryos and natural E6.5, E8.5 embryos to perform the single‐cell RNA‐seq by 10X Genomics (Figure 6c–f). All the filtered cells from E6.5, E8.5, PDCO, and PD embryos could be clustered into 15 categories, including Allantois, Cardiomyocytes, Endothelium, Epiblast, Erythroid, ExE ectoderm, ExE endoderm, Forebrain/Midbrain/Hindbrain, Gut, Mesenchyme, Neural crest, Neuromesodermal progenitors, Paraxial mesoderm progenitors, Parietal endoderm, Surface ectoderm (Figure 6c). Notably, we found the cardiomyocytes and gut only could be detected in E8.5 and PDCO embryos, and the Endothelium percentage of PDCO embryos was more comparable with E8.5 embryos, both of which indicated that the PDCO embryos shared features with E8.5 embryos and could develop to early organogenesis stage beyond gastrulation (Figure 6e). Second, compared with E6.5 or PD embryos, E8.5 and PDCO embryos possessed lower proportion of the extraembryonic tissues (including EXE ectoderm and EXE endoderm), suggesting the embryos cultured on PDCO could self‐organize and balance the development of intra‐/extra‐embryonic tissues (Figure 6e). In contrast, the embryos cultured on PD exhibited overgrowth on extraembryonic tissues, which might impact the development of intraembryonic tissues (Figure 6e). Actually, we cannot deny that the percentage of Surface ectoderm in PD and PDCO embryos were lower than the E8.5 counterpart, implying that the differentiation of ectoderm in IVC embryos displayed slight distinct from natural embryos (Figure 6e). Collectively, PD embryos possessed a limited proportion of advanced cell types or definitive organ cells, while PDCO embryos exhibited more advanced developmental features and their cell component was more similar with E8.5 natural embryos (Figure 6f).

### 2.6 Discussion

The current in vitro culture of mouse embryos could support E3.5 embryos to E6.75,^[^ ^18^, ^25^ ^]^ E4.5 embryos to E6.0,^[^ ^14^, ^15^ ^]^ and E5.5 embryos to E11.0.^[^ ^16^ ^]^ It seems that in vitro embryo development at the preimplantation stage is rather difficult. Here, we have established a uterus‐inspired culture system for embryo invasion and postimplantation embryo development by mimicking the uterine microenvironment from the mechanical properties and matrix microstructures points of view. In the UN system, we can clearly observe the special interaction between the embryo and the UN system, indicated by the trophoblast cells invasion into the collagen, which was remodeled under the action of the embryo. The UN system enables studies on the physical mechanism of communications between the embryos with the culture substrates.^[^ ^5^, ^44^ ^]^ The evidence that the trophoblasts of the PDCO embryos have lower modulus than those of the PD embryos (Figure 4g,h) suggests that the substrates can affect the physical nature of the embryos, which endows the embryo with different capacity of invasion into the substrates and further development.^[^ ^41^ ^]^ The thickness of thin collagen layer for PDCO is 46 ± 10 µm and Figure 4c shows that the trophoblast cells of the embryo invade into the collagen, so the trophoblast cells may feel the stiffness of PDMS, which may activate the YAP (Yes associated protein) cell pathway to regulate embryonic development. Whereas the thickness of thick collagen layer for PDCO‐T is 417 ± 137 µm, and the trophectoderm cells may only feel the stiffness of collagen, which may not activate the YAP pathways.^[^ ^45^, ^46^, ^47^ ^]^ Similarly, direct exposure of embryos to PDMS from day one may cause rapid activation of the YAP cell pathway to promote trophoblast expansion (Figure 4d–f). Physical factors affecting embryonic development will be an important direction for our follow‐up research. Although in vivo embryo implantation is a complex process, this UN system can provide a platform for future research on embryo implantation from the aspects of physicochemical properties and compositions.

Dramatic changes in embryonic structure and cellular differentiation make it challenging to prolong normal embryo development and to portray the precise status of the postimplantation embryo in vitro. The UN system provides a platform to studying postimplantation embryonic developmental events, such as egg cylinder formation (Figure 3), the development of blood cells and blood vessels at the yolk (Figure S15f,g, Supporting Information), the occurrence and migration of primitive streak (Figure 5c), the development of yolk sac, which could broaden our knowledge of embryogenesis from implantation to gastrulation stages (Figure 6). Besides, the IVC embryos really have differences in the size, cellular component, developmental clock, morphology, and molecular features from natural embryos and delayed development with natural embryos, which might result from the dramatic differences in the developmental environment between in vivo and in vitro. We believe, with the gradually filling of the gap on the knowledge about the natural embryonic developmental microenvironment, especially the physicochemical property and the extraembryonic tissue features, the in vitro cultures system will be further optimized with combination of cells and biomaterials, which will support the embryonic development to more advanced stage with more representative and comparable biological characteristics.

## 3 Conclusion

In summary, our study has built up a robust embryo culture system inspired by the mechanical properties and microstructures of the uterus, extending embryo culture to the early organogenesis stage, enabling to provide powerful tools for in‐depth studies of embryonic implantation in the future. For further investigation, this platform could be a helpful tool for developmental biology, biomaterial science, and tissue engineering.

## 4 Experimental Section

### Animal and Ethical Approval

ICR 7‐ to 8‐week‐old females and 8‐week‐old males were purchased from SPF (Beijing) Biotechnology. The ICR mice were raised under specific‐pathogen‐free (SPF) conditions and handled following the guidelines of the Animal Care and Use Committee of the Institute of Zoology, Chinese Academy of Sciences (Ethical approval No. IOZ20180058).

### Scanning Electron Microscopy

At 4 °C, the natural uterus, decell uterus, and collagen were fixed for 4 h in 2.5% glutaraldehyde. Then, the samples were dehydrated for 15 min each in 30, 50, 75, 80, 90 vol% ethanol and 45 min in 100 vol% ethanol. After supercritical drying (aotosamdri‐815, Tousimis), the samples were sprayed with platinum (JEC‐3000FC, JEOL) for SEM observation (SU8010, Hitachi, Japan) and Field Emission Scanning Electron Microscopy (FE‐SEM) observation (SUPRA55, CARL ZEISS, Germany).

### SEM Movies

In order to clearly show the process of observing SEM images and continuously display microstructures at the large‐scale, the SEM movies of the step‐by‐step magnification and microstructures at the large scale were made. The step‐by‐step magnification videos were made as follows. First, FE‐SEM images (SUPRA55, CARL ZEISS, Germany) with progressive magnification such as 50 X, 100 X, 200 X, 400 X, 800 X, 1600 X, 3200 X, 6400 X, 12 800 X, and 25 600 X. In order to achieve the movies of the step‐by‐step magnification at the same position, all the images were got in the same place. Among them, the smallest magnification is used to display the samples in a large field of view, and the selection of the smallest magnification will be different according to the sizes of the samples. The SEM images are at high resolution with 2.8 nm pixels, which can be used for an enlarged display of images. The images were expanded by the bicubic interpolation method. The pixel value *p(x,y)* of the new intermediate surface is obtained by calculating the relationship between the point to be interpolated *p(x,y)* and the surrounding 16 pixels (4 × 4), as shown in the following equation^[^ ^48^ ^]^

(1) $$p\left(x,y\right)=\sum_{i=0}^{3}\sum_{j=0}^{3}a_{ij}x^{i}y^{j}$$

The 16 coefficients *a_ij_* in the formula need to be determined, where *i* and *j* are the orders of the polynomials in the *x* and *y* directions, respectively.

When the image is enlarged to the adjacent magnification, the image of the next magnification is used instead, and so on, until the maximum magnification is reached. The scale bar is introduced according to the pixel size, and the magnification is displayed simultaneously. SEM movies of microstructures at the large‐scale are made as follows. First continuous SEM images in a rectangular area with 2.8 nm pixels and adjacent images maintain 16% overlap were got. The following methods were used to stitch images, including Scale Invariant Feature Transform (SIFT), Random Sample Consensus,^[^ ^49^ ^]^ and linear transformation. These images were put together into one figure using ImageJ. In the overall figure at low magnification, when the indicator box gradually moved, the corresponding enlarged image is displayed simultaneously realized. See the appendix for specific MATLAB codes for the SEM movies.

### Sectional SEM Observation

This experiment is to get the SEM images of embryos invading into the collagen fibers. The samples of embryos in UN on IVC day 2 were placed on ice and washed three times in phosphate buffered saline (PBS) buffer on a shaker. Then, 4% (PFA, Sigma) and 2.5% glutaraldehyde (GA, Sigma) fixatives were prepared in 0.1 m PBS, pH 7.2, and the samples were fixed in fixatives at room temperature for 1 h and then overnight at 4 °C (the volume of the fixative was more than ten times that of the fixed material). The sample was rinsed 3 times in 0.1 m PBS (pH 7.2) and treated with 2% osmic acid in 0.1 m PBS (pH 7.2) overnight at 4 °C. Gradient dehydration was performed in 50%, 70%, 80%, 90% ethanol/water solution for 10 min each, 100% ethanol for 10 min, 50% ethanol, and 50% acetone for 10 min, and 100% acetone for 10 min for three times. Acetone/resin solutions at ratios of 1:1, 1:2, and 1:3 were applied for 4 h each, and pure resin was used overnight under negative pressure. The catalyst was added immediately before resin use; otherwise, it could be stored at 4 °C for a long time. The samples were cured at 37 °C for 12 h, 45 °C for 12 h, and 60 °C for 1d. The sample was sliced. The slices were placed in an oven at 60 °C for 30 min and then stored at room temperature. The samples could be used for backscattered electron imaging (SEM, GeminiSEM300, Carl‐Zeiss Microscopy GmbH, Oberkochen, Germany) to observe the phenomenon of the embryos invading into the collagen fibers.

### Uniaxial Tension Test

At least ten samples were prepared and tested, including uterine horn and PDMS samples. The test equipment types used were a Mark‐10 ESM303 (Mark‐10 Corporation, NY) and an Instron 5943 Tensile tester (Instron, Canton, MA). Clamps were used to fix both ends of the sample. The inner diameter d and outer diameter D of the samples for the uterine horn samples were measured. The width W and thickness T of the sample for the PDMS samples were measured. Before starting the experimental measurement, the initial length l of the sample between the clamps was measured. The load was applied at a 10 mm min^−1^ rate until the sample broke. The force *F* during the experiment and the change in length *∆l* of the sample was recorded. Then, the stress *σ = F*/*A* during the experiment was obtained, where A is the cross‐sectional area of the sample. The calculation formulas for the uterine horn and PDMS were as follows: *A = π*(*D* ^2^ *‐d* ^2^)/*4* and *A = WT*. The strain was calculated as follows: *ε = ∆l*/*l*_0_. The initial linear slope of the stress–strain curve was used to calculate the tensile elastic modulus (Young's modulus, *E*) (from 10% to 40% strain). All these factors could be combined into one equation for *F*

(2) $$F=E\frac{\Delta L}{L_{0}}A$$

### Atomic Force Microscopy Test

AFM (Nanowizard4, Bruker, USA) was used to test the elastic modulus of the uterus layer by layer, including the endometrium and the myometrium and the method has been widely used in biomaterials and cell property characterization.^[^ ^50^ ^]^ AFM measurements were performed using silicon nitride cantilevers with a nominal spring constant of 0.12 N m^−1^ (NP‐O10, Nanoworld, USA) and a 30 µm diameter silica bead (Thermo). Each sample was measured at 5 points, and 8 force curves were collected at each end. The applied mechanics and Hertz models could be used for data fitting and calculation with the spherical indenters involved in the indentation. The key factors have been specified in Equations ((3), (4), (5), (6)). Following the Hertz model describing the indentation of elastic bodies, the force‐to‐penetration equation is

(3) $$F=\frac{4}{3}\sqrt{R}\delta^{3/2}E^{*}$$

here, *F* is the applied force, *R* is the radius of the indenter tip, *δ* is the indentation depth, and E* is the reduced modulus, which can be defined by the combination of the plane strain moduli of the indenter (*E*_1_) and the substrate modulus (*E*_2_)^[^ ^51^ ^]^

(4) $$\frac{1}{E^{*}}=\frac{1-v_{1}^{2}}{E_{1}}+\frac{1-v_{2}^{2}}{E_{2}}$$

where *v*_1_ and *v*_2_ are the Poisson's ratios of the indenter and the substrate, respectively, and the indenter is much stiffer than the substrate (*E*_1_ >> *E*_2_), so the reduced modulus E* is simply

(5) $$E^{*}=\frac{E_{2}}{1-v_{2}^{2}}$$

For uteri, the Poisson ratio (*v*_2_) is assumed to be 0.5^[^ ^52^ ^]^ Equations ((3), (4), (5)) become

(6) $$F\approx \frac{16}{9}E_{2}\sqrt{R}\delta^{3/2}$$

The data in the loading section of the load‐displacement curve were used to determine Young's modulus using a fit of all data points from the contact point to the maximum load point.

### Fabrication of the UN System

The mass ratio of the matrix and crosslinker in the PDMS prepolymer (Sylgard 184, Dow Corning, USA) was 10:1. The prepolymer was blended uniformly and poured over a four‐well plate (176 740, Thermo). PDMS prepolymer (0.2 g) was added per well, and the PDMS wells were cured at 80 °C for 2 h and processed by plasma for 30 W for 5 min. The PDMS surface was pretreated with 0.2 mg mL^−1^ sulfo‐SANPAH and coated with 50 µg mL^−1^ Collagen I for 10 h. Dialysis of Collagen I was added to the coated PDMS wells, and they were cured at 37 °C for 1 h. For collagen thickness measurements, phenolphthalein (P816041‐25 g, Macklin) was added to the dialysis fluid (PBS), adding color to Collagen I for easy identification. The collagen after dialysis was added to the wells coated with PDMS, and cured at 37 °C for 1 h. 12 cured samples were quickly cut from the middle by a sharp knife. The cross sections were imaged under the microscope. ImageJ was used for analyzing the collagen thickness.

### Embryo Recovery and Culture

Pregnant mice were humanely culled at 3.5 days post‐coitum through cervical dislocation, and the embryos were flushed out with a flush medium and seeded onto a specific well plate. This time was recorded as IVC day 0. Embryo culture was performed at 37 °C in a 5% CO_2_ atmosphere.

### In Vitro Culture Medium

Flush medium: CMRL 1066 (11 530 037, Invitrogen) + 5 × penicillin‐streptomycin (60162ES76, YEASEN) + 10% FBS (SE200‐ES, Vistech).

Medium I for embryos on IVC days 0–1: CMRL 1066+ 1 × penicillin‐streptomycin+ 1 × GlutaMAX Supplement (35 050 061, Thermo) + 1 × MEM Non‐Essential Amino Acids Solution (11 140 050, Thermo) + 0.5 × N‐2 Supplement (17 502 048, Gibco) + 0.5 × B‐27 Supplement (17 504 044, Gibco) + 10% FBS.

Medium II for embryos on IVC day 2: CMRL 1066 + 1 × penicillin‐streptomycin + 1 × GlutaMAX Supplement+ 1 × MEM Non‐Essential Amino Acids Solution + 0.5 × N‐2 Supplement + 0.5 × B‐27 Supplement + 20% FBS.

Medium III for embryos on IVC days 3–4: CMRL 1066 + 1 × penicillin‐streptomycin + 1 × GlutaMAX Supplement + 1 × MEM Non‐Essential Amino Acids Solution + 30% KnockOut Serum Replacement (10 828 028, Gibco).

Medium IV for embryos on IVC day 5: CMRL 1066 + 1 × penicillin‐streptomycin+ 1 × GlutaMAX Supplement + 1 × MEM Non‐Essential Amino Acids Solution + 30% KnockOut Serum Replacement + 30% RS (rat serum) + 0.5 mg mL^−1^ glucose (Sigma, D9434).

Medium V medium for embryos on IVC days 6–10: CMRL 1066+ 1 × penicillin‐streptomycin + 1 × GlutaMAX Supplement + 1 × MEM Non‐Essential Amino Acids Solution + 50% RS + 1 mg mL^−1^ glucose

### Whole‐Mount Immunofluorescence Staining and Imaging of Mouse Embryos

To characterize embryos on PD and PDCO from IVC day 1 to day 5 and in vivo embryos recovered at E4.5‐E6.5 were characterized. The embryos were fixed overnight with 4% PFA at 4 °C; permeabilized for 1 h in 1% Triton X‐100 in PBS at 37 °C; blocked for 1 h in 0.1% Tween‐20, 0.01% Triton X‐100, and 1% BSA in PBS at 37 °C; and incubated with primary antibodies at 4 °C overnight. The primary antibodies were OCT4 (1:100, sc‐8629, Santa Cruz), F‐actin (1:100, ab205, Abcam), CDX2 (1:100, 3977S, Cell Signaling Technology), Collagen I (1:100, ab90395, Abcam), FOXA2 (1:100, 8186S, Cell Signaling Technology), TFAP2C (1:100, sc‐12762, Santa Cruz), SOX2 (1:100, ab97959, Abcam), EOMES (1:100, ab23345, Abcam), T (1:100, ab209665, Abcam), and Phall (1:500, 40737ES75, Yeasen). The following secondary antibodies were incubated for 2 h at room temperature: Alexa 488 donkey anti‐mouse (1:500, A21202, Invitrogen), Cyanine3 goat anti‐mouse (1:500, A10521, Invitrogen), Alexa Fluor 647 goat anti‐mouse (1:500, A21235, Invitrogen), Alexa 488 goat anti‐rabbit (1:500, A11034, Invitrogen), and Cyanine3 goat anti‐rabbit (1:500, A10520, Invitrogen). Nuclear staining and incubation were performed for 10 min at room temperature in 10 µg mL^−1^ Hoechst 33 342 (H3570, Invitrogen). The embryos were imaged on a Leica SP8 Zeiss LSM780 confocal microscope. Imaris 9.0.2 software was used to construct the 3D images.

### Identification of Embryos on PDCO from IVC day 6–day 10 and In Vivo Embryos at E7.5‐E8.5

Embryos were fixed overnight with 4% PFA at 4 °C; permeabilized for 10 h in 2% Triton X‐100 in PBS at room temperature; blocked overnight in 0.1% Tween‐20, 0.01% Triton X‐100, and 1% BSA in PBS at 4 °C; and incubated with primary antibodies at 4 °C overnight. The primary antibodies were PAX6 (1:100, AF8150, R&D Systems), CD31 (1:100, ab222783, Abcam), GATA4 (1:100, sc‐25310, Santa Cruz), myosin light chain (1:100, ab79935, Abcam), myosin heavy chain (1:100, 53‐6503‐82, eBioscience). The secondary antibodies were incubated overnight at 4 °C. The secondary antibodies were Alexa 488 goat anti‐rabbit (1:500, A11034, Invitrogen) and Alexa Fluor 647 donkey anti‐sheep (1:500, A21448, Invitrogen). Nuclear staining and incubation in were performed 10 µg mL^−1^ Hoechst 33 342 (H3570, Invitrogen) for 10 min at room temperature. The embryos were imaged on confocal microscopes Leica SP8, and Zeiss LSM780 and Imaris 9.0.2 software was used to construct the 3D images/videos.

### Preparation of the Decell Uterus

Uterine perfusion was performed through the aorta (flow rate = 2 mL min^−1^) with 1% sodium dodecyl sulfate, 0.1% sodium dodecyl sulfate, 0.01% sodium dodecyl sulfate, and 1% Triton X‐100, 0.1% Triton X‐100, and 0.01% Triton X‐100 for 24 h, respectively. And then with water for 24 h, the decell uterus was fixed.

### Single‐Cell Isolation and Transcriptome Library Construction

Single‐cell isolation and transcriptome library construction were performed according to a protocol described previously.^[^ ^26^ ^]^ Single cells from embryos of PD and PDCO on IVC day 2 were harvested to generate RNA‐seq data and compared to the in vivo embryos of E4.5 for analysis. The raw files were processed with Cell Ranger using the default mapping arguments. Gene expression analyses and cell‐type identifications were performed using Seurat V3.1. The data were merged and normalized to published data using the IntegrateData function,^[^ ^38^ ^]^ as described in the Seurat vignettes. The tSNE was used for nonlinear dimensional reduction. The figures were produced in Seurat using the DimPlot and VlnPlot functions.

### RNA‐seq Library Preparation and Data Analyses

Total RNA was extracted from the collected samples with a PureLink RNA Mini Kit (Life). The RNA‐seq libraries were prepared with the NEBNextUltraTM RNA Library Prep Kit for Illumina. Sequencing was performed on an Illumina HiSeq X‐Ten sequencer with a 150 bp paired‐end sequencing reaction. The clean reads were analyzed with Hisat2 (version 2.1.0) and StringTie using the mm10 annotation and default settings. Reads with unique genome locations and genes with no less than 1 FPKM in at least one sample were used for the next step of the analyses. The threshold for filtering differentially expressed genes was set at twofold: increased or decreased expression that was less than twofold was not considered significant. Tissue lineage marker genes were downloaded from https://marionilab.cruk.cam.ac.uk/MouseGastrulation2018/, and heatmap analyses were performed with hierarchical clustering and heatmap.2 functions in R. GO analyses were performed using DAVID online.

### Statistical Analyses

One‐way ANOVA with Welch's correction statistically analyzed the collected data if sample sizes were unequal and with the Bonferroni post hoc test or two‐tailed Student's *t*‐test in software GraphPad Prism 7 for Window and graphed in software Origin 9. The data are represented as the mean ± s.d. Figure legends indicate the number of independent experiments performed in each analysis. All experiments were repeated at least three times unless otherwise noted. In box plots, the center lines are the median values of the data. The box limits (interquartile range (IQR)) represent the data ranges between the 25th percentile (the first quartile, Q1) and the 75th percentile (the third quartile, Q3). The upper and lower whiskers represent Q3 + 1.5 × IQR and Q1 – 1.5 × IQR, respectively.

## Conflict of Interest

The authors declare no conflict of interest.

## Author Contributions

Z.G., J.G., J.Z., and G.F. contributed equally to this work. Q.G. conceived and designed the project together with Z.G., S.T.W., and H.M.W.; Z.G., J.G., and J.L.Z. equally designed and performed all experiments and prepared the manuscript; G.H.F., X.N.W., Z.L.G., L.Y.J., Y.Q.W., L.W., J.H., and W.L. provided assistance in carrying out experiments and discussed results. K.L., S.J., L.Y.W., Y.H.X., X. C., Y.M.W., S.S.G., M.Y., L.L.L., and H.H. performed experiments during the manuscript revision. Z.G., J.G., and J.L.Z., together with S.T.W., H.M.W., and Q.G., discussed results and prepared the manuscript.

## Supporting information

Supporting Information

Supplemental Movie 1

Supplemental Movie 2

Supplemental Movie 3

Supplemental Movie 4

Supplemental Movie 5

Supplemental Movie 6

Supplemental Movie 7

## Data Availability

The data that support the findings of this study are available from the corresponding author upon reasonable request.
