# Supplementary material for: A Uterus‐Inspired Niche Drives Blastocyst Development to the Early Organogenesis
Source: Adv Sci (Weinh). 2022 Jul 17;9(28):2202282. doi: 10.1002/advs.202202282 (PMC9534964; doi:10.1002/advs.202202282)
Supplement: Supplementary file 1 — Supporting Information [file ADVS-9-2202282-s006.pdf]

ADVANCED SCIENCE
Open Access

Supporting Information

for *Adv. Sci.*, DOI 10.1002/advs.202202282

A Uterus-Inspired Niche Drives Blastocyst Development to the Early Organogenesis

Zhen Gu, Jia Guo, Jinglei Zhai, Guihai Feng, Xianning Wang, Zili Gao, Kai Li, Shen Ji, Leyun Wang, Yanhong Xu, Xi Chen, Yiming Wang, Shanshan Guo, Man Yang, Linlin Li, Hua Han, Liyuan Jiang, Yongqiang Wen, Liu Wang, Jie Hao, Wei Li, Shutao Wang, Hongmei Wang* and Qi Gu**

Supplementary information for

A uterus-inspired niche drives blastocyst development to early organogenesis

Zhen Gu, Jia Guo, Jinglei Zhai, Guihai Feng, Xianning Wang, Zili Gao, Kai Li, Shen Ji, Leyun Wang, Yanhong Xu, Xi Chen, Yiming Wang, Shanshan Guo, Man Yang, Linlin Li, Hua Han, Liyuan Jiang, Yongqiang Wen, Liu Wang, Jie Hao, Wei Li, Shutao Wang, Hongmei Wang,* and Qi Gu**

J. Guo, Dr. J. Zhai, Prof. G. Feng, X. Wang, Z. Gao, K. Li, Dr. S. Ji, Dr. L. Wang, Y. Xu, Y. Wang, S. Guo, Prof. L. Jiang, Prof. L. Wang, Prof. J. Hao, Prof. W. Li, Prof. H. Wang, Prof. Q. Gu

State Key Laboratory of Membrane Biology, The State Key Laboratory of Stem Cell and Reproductive Biology, Institute of Zoology, Chinese Academy of Sciences, Beijing 100101, P. R. China

E-mail: qgu@ioz.ac.cn; wanghm@ioz.ac.cn

Dr. Z. Gu, Prof. Y. Wen

Department of Chemistry and Biological Engineering, University of Science and Technology Beijing, Beijing 100083, P. R. China

J. Guo, Dr. J. Zhai, Prof. G. Feng, X. Wang, Z. Gao, K. Li, Dr. S. Ji, Dr. L. Wang, Y. Xu, Y. Wang, S. Guo, Prof. L. Wang, Prof. J. Hao, Prof. W. Li, Prof. H. Wang, Prof. Q. Gu

Beijing Institute for Stem Cell and Regenerative Medicine, Beijing 100101, P. R. China

Dr. Z. Gu, Dr. M. Yang, Prof. S. Wang

CAS Key Laboratory of Bio-inspired Materials and Interfacial Science, Technical Institute of Physics and Chemistry, Chinese Academy of Sciences, Beijing, 100190, P. R. China.

E-mail: stwang@mail.ipc.ac.cn.

J. Guo, Dr. J. Zhai, Prof. G. Feng, Z. Gao, K. Li, Dr. L. Wang, Y. Xu, Y. Wang, S. Guo, Prof. L. Wang, Prof. J. Hao, Prof. W. Li, Prof. S. Wang, Prof. H. Wang, Prof. Q. Gu
University of Chinese Academy of Sciences, Beijing 100049, P. R. China.

Prof. L. Jiang
School of Life Sciences, Northeast Agricultural University, Harbin, 150030, P. R. China.

Prof. X Chen, Prof. L Li, Prof. H Han
National Laboratory of Pattern Recognition, Institute of Automation, Chinese Academy of Sciences, Beijing, 100190, P. R. China.

Keywords: Uterus-inspired, implantation, *in vitro* culture (IVC), early organogenesis

Table of Contents:

Supplementary notes

Supplementary Figures 1 to 17 with legends

Supplementary Tables 1 to 3

Legends for Supplementary Movies 1 to 7

Supplementary references

Supplementary notes

Previous studies on embryo culture and embryo-material interactions

The first mouse blastocyst cultured *in vitro* was reported by being injected into the fiber network of the bovine eye.[1] Currently, the typical dishes used for embryo culture are made of polystyrene, silicone, or specialized plastic polymer-coated with collagen, laminin, fibrinogen, or some other matrix[2-6] and the embryos exhibited limited developmental potency.

The cell microenvironment, a complicated and dynamic biochemical and physical environment, can control cell behaviors and development and determine cell fates.[7] An embryo, which is a well-organized cell cluster, is also sensitive to the surrounding conditions.[8,9]

3D biomaterial systems have provided a paradigm shift in cell culture *in vitro,* and considerable research efforts have been devoted to material properties, including macro/nanostructure, elasticity, stiffness, and micro/nanopatterns that influence cell states[10-14] through chemical pathways.[15-20]

Our UN platform was established according to a good understanding of the structure of the uterus and developmental biology. Developing an appropriate culture method *in vitro* is crucial to exploring the black box of embryo development and stem cell-based embryo assembly, which has attracted much attention.[6,21-29] To the best of our knowledge, the UN system not only extends the embryo culture period from the initial blastocyst stage to the heartbeat-like stage but also reveals molecular regulators between materials and embryos, which govern cell migration, to regulate embryo spreading and developmental potential.[30-35]

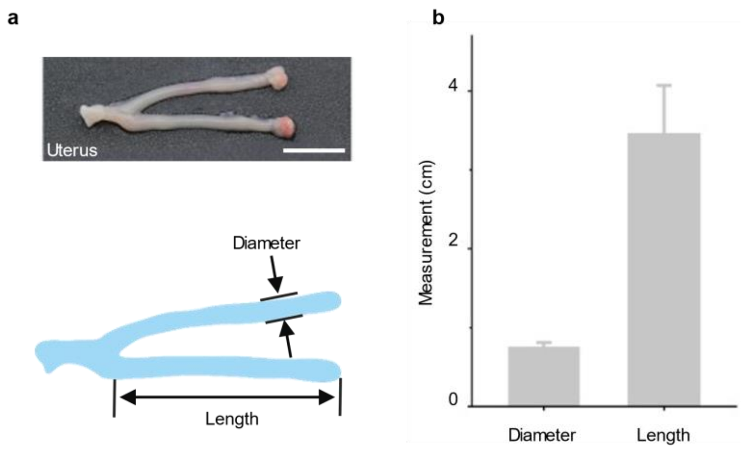

Figure S1. Macroscopic images of the uterus. a) Digital morphology picture of the uterus from the pregnant mouse (E3.5) (upper panel) and a schematic of the uterus (lower panel). Scale bar, 1 cm. b) The statistics of the diameter and length of the uterine horn. n = 6 uterine horns of 3 mice.

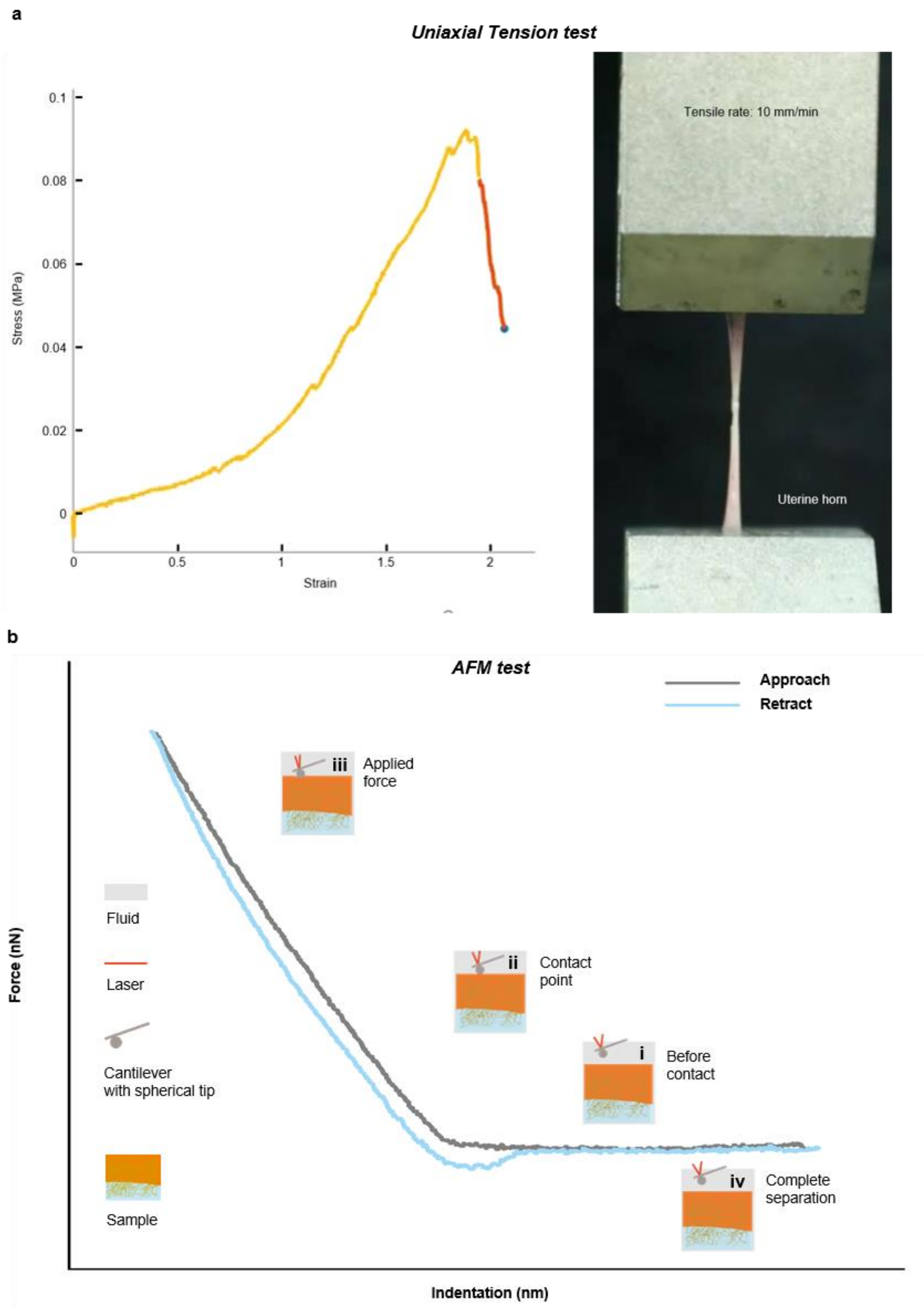

Figure S2. Evaluation of mechanical properties using three methods. a) A screenshot of representative stress-strain curve for the uniaxial tensile test using a universal testing machine and the points of the stress-strain curve (left panel) corresponding to the actual stretching process of the uterine horn (right panel) taking the uterus sample as an example. b) Schematic

diagrams of AFM test in which the cantilever approaches the sample and then withdraws and force distance curves measuring the deflection of the cantilever.

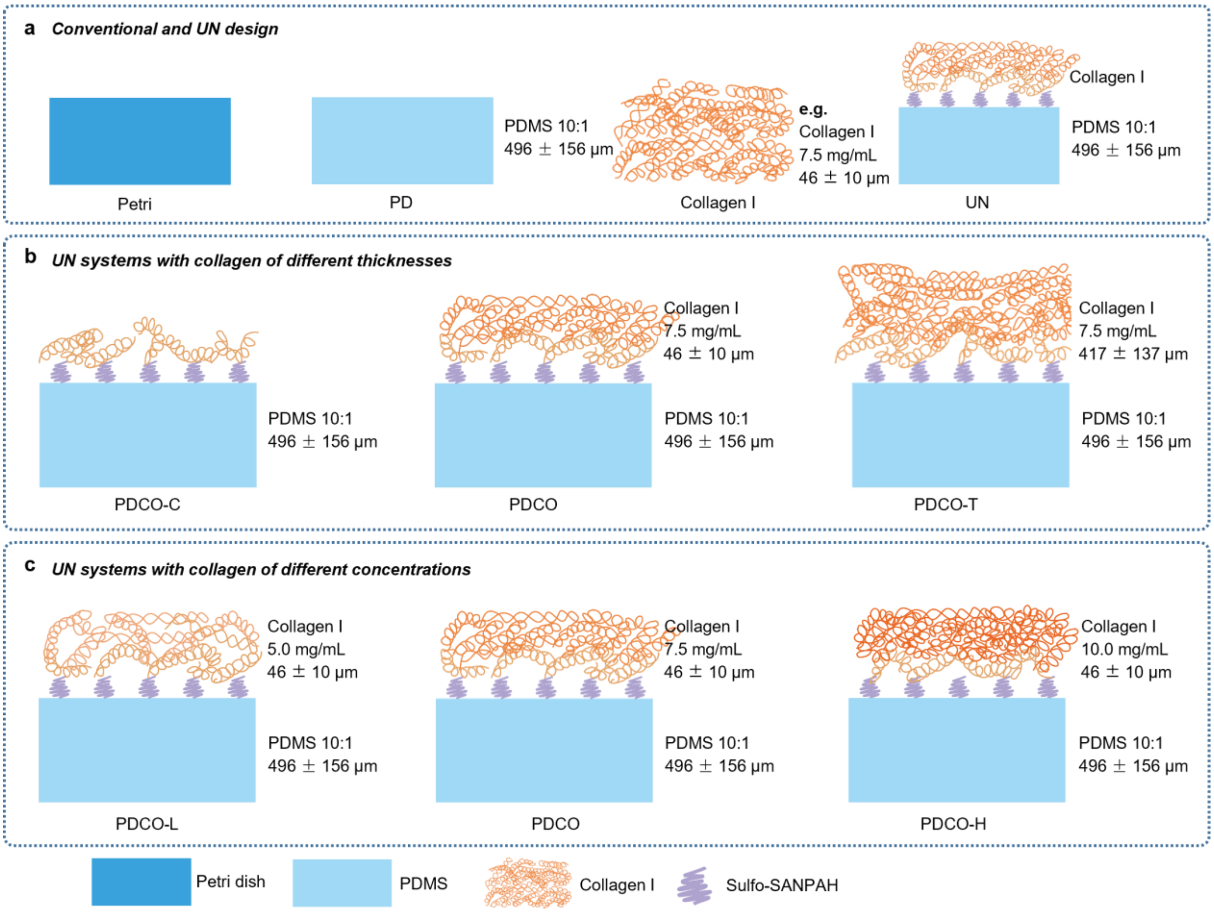

Figure S3. Schematic diagram of the UN systems for embryo culture *in vitro*. a) Conventional systems (Petri, PD and CO) and UN system. Petri: Nunc 4-well Petri dish. PD: PDMS with a thickness of 496 ± 156 μm. CO: Collagen I. i. UN: the designed system with bottom layer PDMS (496 ± 156 μm in thickness) and top layer collagen of different thicknesses and concentrations detailed in middle and bottom panels. b) The UN systems with different thicknesses of collagen gel. PDCO-C: PDMS (496 ± 156 μm thick) coated with collagen. PDCO: bottom layer PDMS (496 ± 156 μm thick) bonded with the top layer collagen (type I, 7.5 mg/mL, 46 ± 10 μm thick). PDCO-T: bottom layer PDMS (496 ± 156 μm thick) bonded with the top layer collagen (7.5 mg/mL, 417 ± 137 μm thick). c) UN systems with different concentrations of collagen. PDCO-L, PDCO and PDCO-H fabricated with top layer collagen gel (46 ± 10 μm thick) at the concentrations of 5.0 mg/mL, 7.5 mg/mL and 10.0 mg/mL, respectively on bottom layer PDMS (496 ± 156 μm thick).

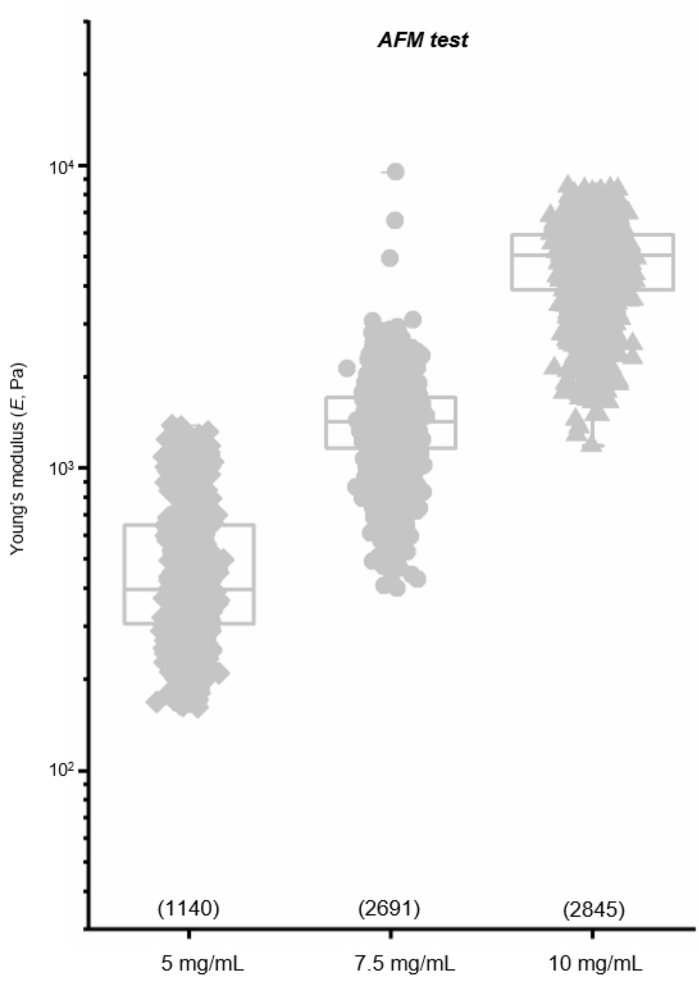

Figure S4. Mechanical properties of collagen at different concentrations. AFM test is used to characterize the mechanical properties as elastic modulus (*E*, Pa) of collagen gels.

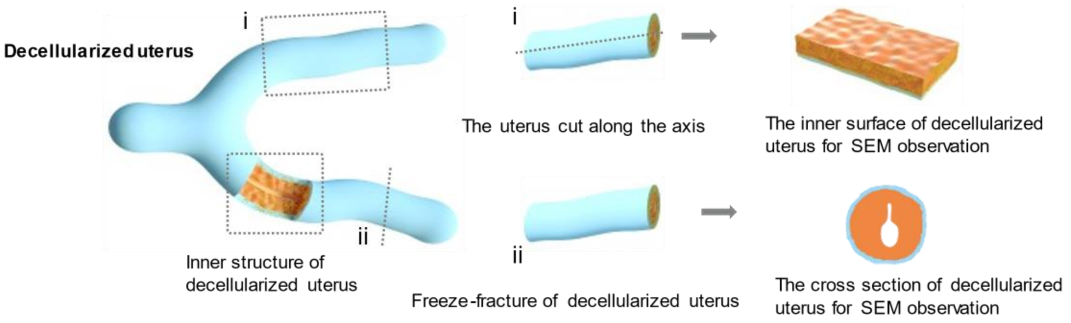

Figure S5. Schematic diagram of the decellularized uterus sample preparation for SEM and FE-SEM observations. The decellularized uterus is cut along the axis of the uterine horn for inner surface observation by SEM (i). The freeze-fractured decellularized uterus is used for cross-sectional SEM observation (ii).

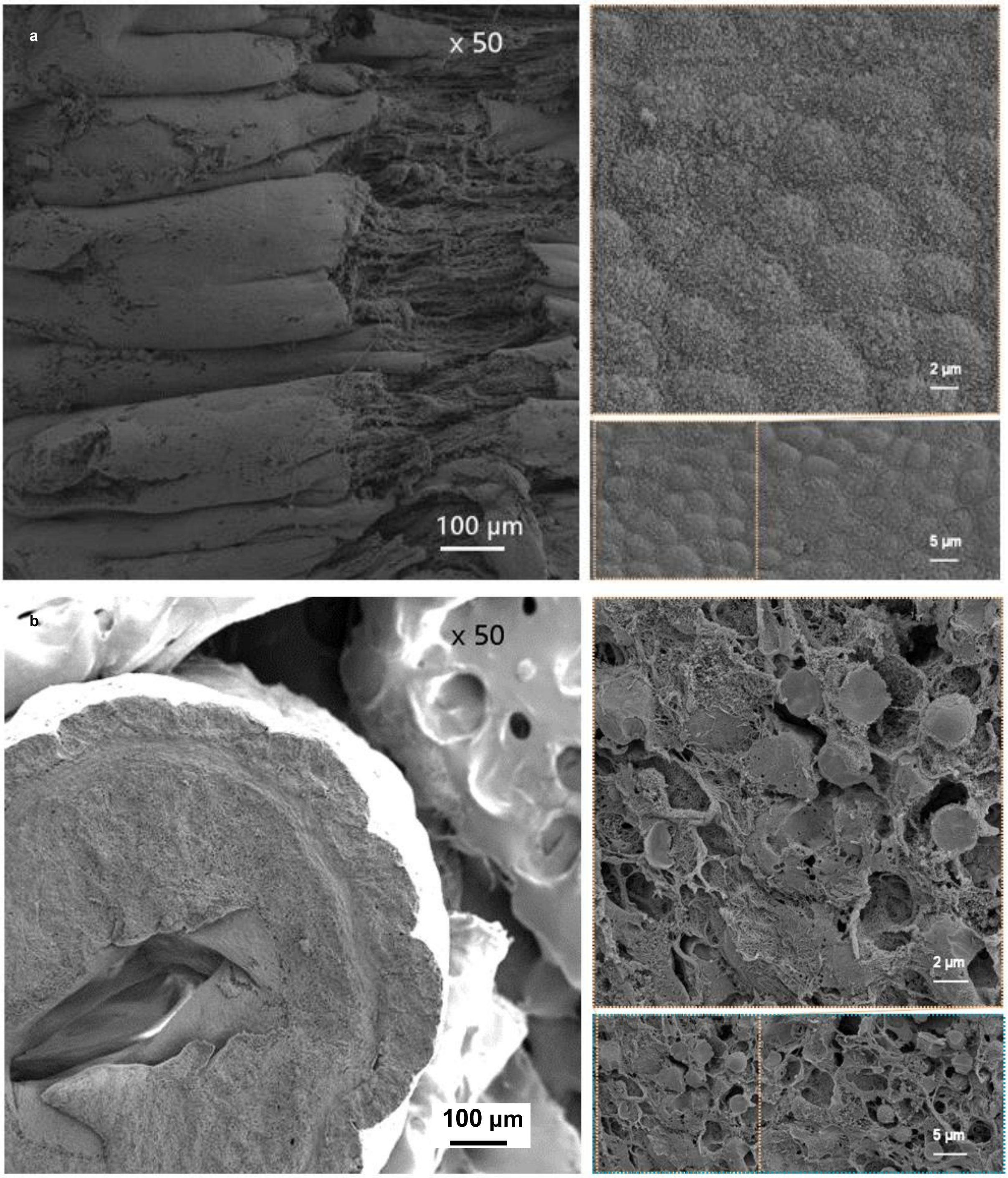

Figure S6. A screenshot of SEM movie for the lumen inner surface and the cross section of the natural uterus (Movies S3 and S4). a) Zooming into the microstructure of uterine lumen inner surface, starting with magnification at 50 × (left panel). An enlarging image is indicated in dotted orange boxes (right panel). b) Zooming into the microstructure of uterine cross-section starting with magnification at 50 × (left panel). An enlarging image is indicated in dotted orange boxes (right panel).

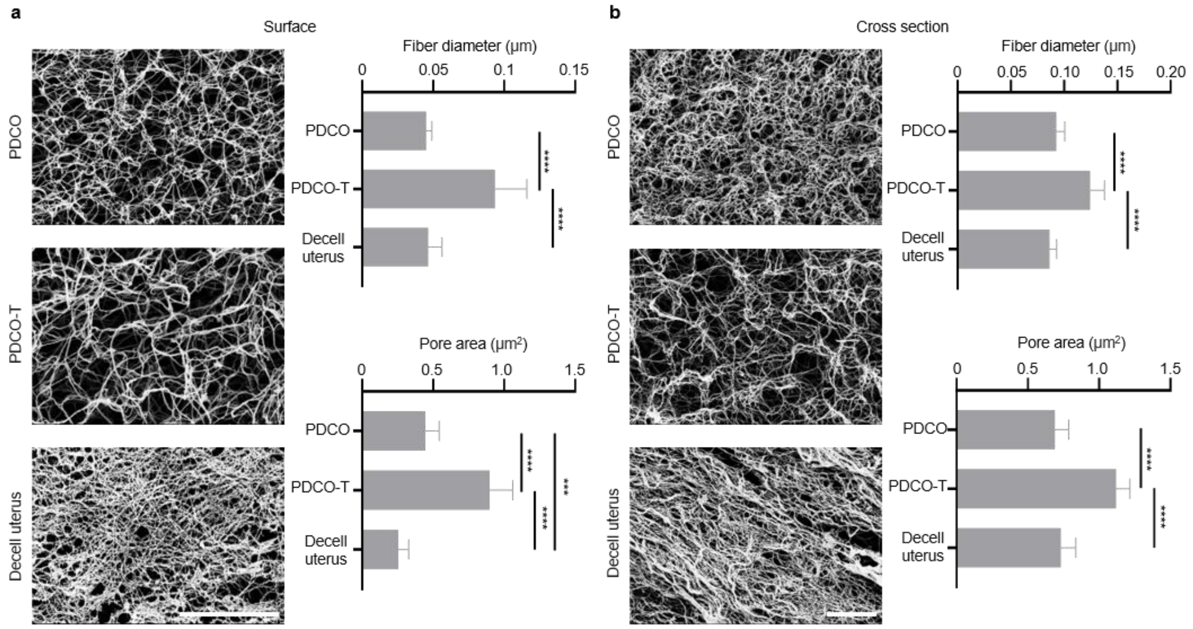

Figure S7. Collagen microarchitecture with different thicknesses. a,b) SEM images of the surfaces and cross-sections of collagen gels with different thicknesses (PDCO and PDCO-T) and the decellularized E3.5 uterus (decell E3.5 uterus), respectively (left panels of a and b). The measurement of the fiber diameter and pore area of the fibrous meshes of the collagen gels and decell uterus based on the SEM images (right panels of a and b). Scale bars, 5μm. Decell E3.5 uterus: n = 3 and 4 from uterus samples for surface and cross section, respectively, 5 images of each sample are counted; PDCO and PDCO-T: n = 3 from collagen gels for cross section and surface, respectively, 5 images of each sample are counted. One-way ANOVA with Bonferroni post hoc test. ns, no significance; ***P < 0.001; ****P < 0.0001.

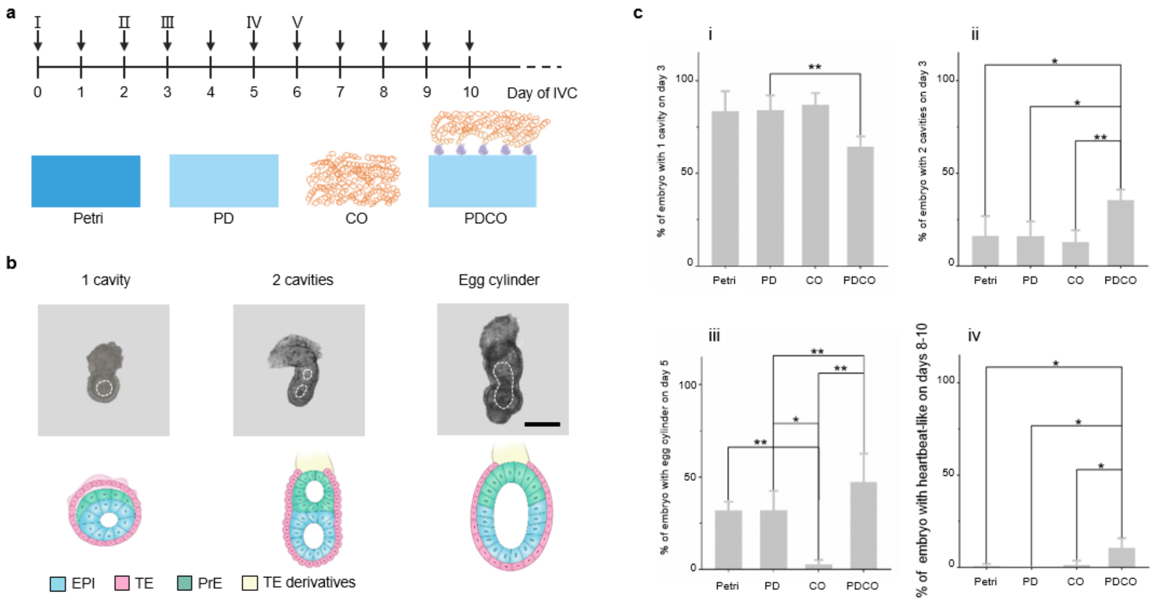

Figure S8. The superiority of PDCO system compared with the conventional culture systems. a) Schematic shows the time course of IVC embryo (top panel) on different systems (bottom panel), with Medium I, II, III, IV and V changing at corresponding time points. b) Top panel, differential interference contrast (DIC) images showing representative IVC embryos development. Scale bars = 100 μm. Bottom panel, schematic illustration of morphological changes of embryos, respectively. c) The statistical analysis of embryo development on different systems (Petri, PD, CO, and PDCO) by the ratios of 1 cavity (i), 2 cavities (ii), egg cylinder formation (iii), and heartbeat-like (iv) on IVC day 3, day 3, day 5 and days 8-10, respectively. All analyses of developmental ratios were normalized by the initial number of embryos seeded on IVC day 0. n = 67, 64, 77, and 71 for Petri, PD, CO, and PDCO, respectively, on IVC day 0. One-way ANOVA with Bonferroni post hoc test. $*P < 0.05$, $**P < 0.01$.

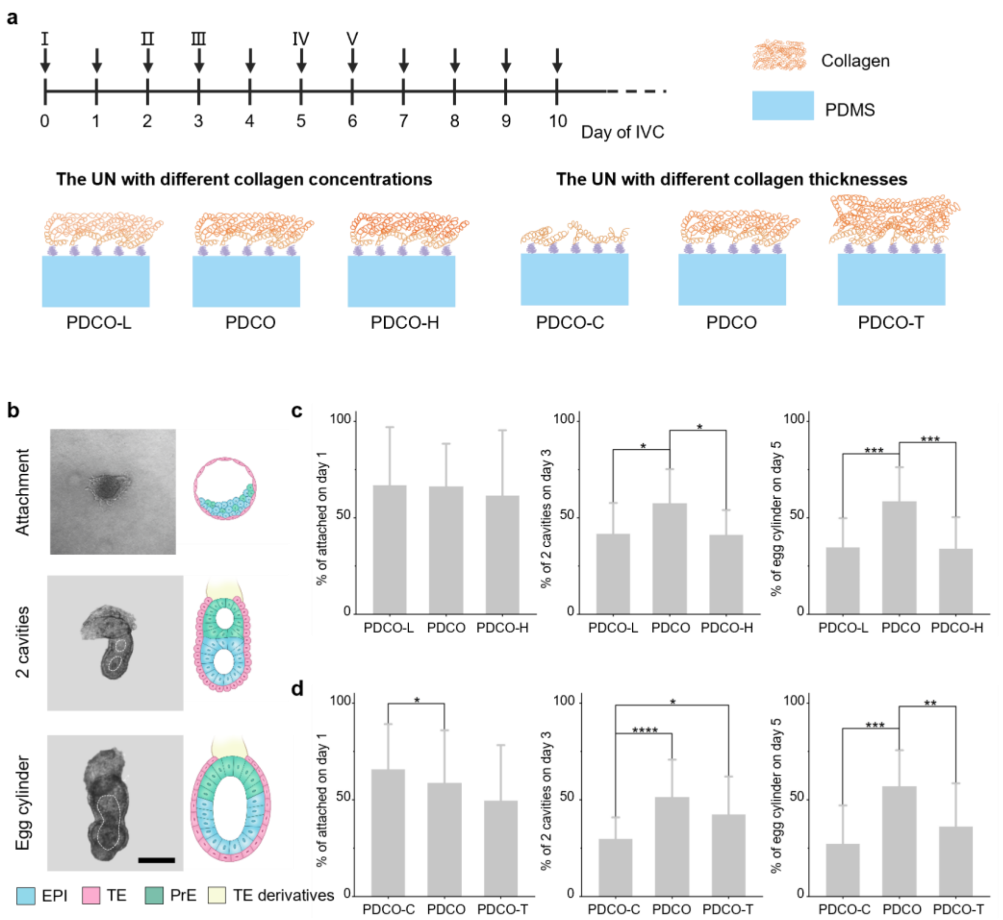

Figure S9. Comparison of embryonic development between different UN systems. a) Schematic shows the time course of IVC embryo (top panel) on different systems (bottom panels), with Medium I, II, III, IV, and V changing at corresponding time points. b) Left panel, differential interference contrast (DIC) images showing representative IVC embryos development. Scale bars = 100 μm. Right panel, schematic illustration of morphological changes of embryos, respectively. c,d) The statistical analyses of embryonic development on the different UN systems. n = 180, 210, 177 for PDCO-L, PDCO, PDCO-H, respectively. n = 123, 174, 168 for PDCO-C, PDCO, PDCO-T, respectively. One-way ANOVA with Bonferroni post hoc test. **P* < 0.05, ***P* < 0.01, ****P* < 0.001.

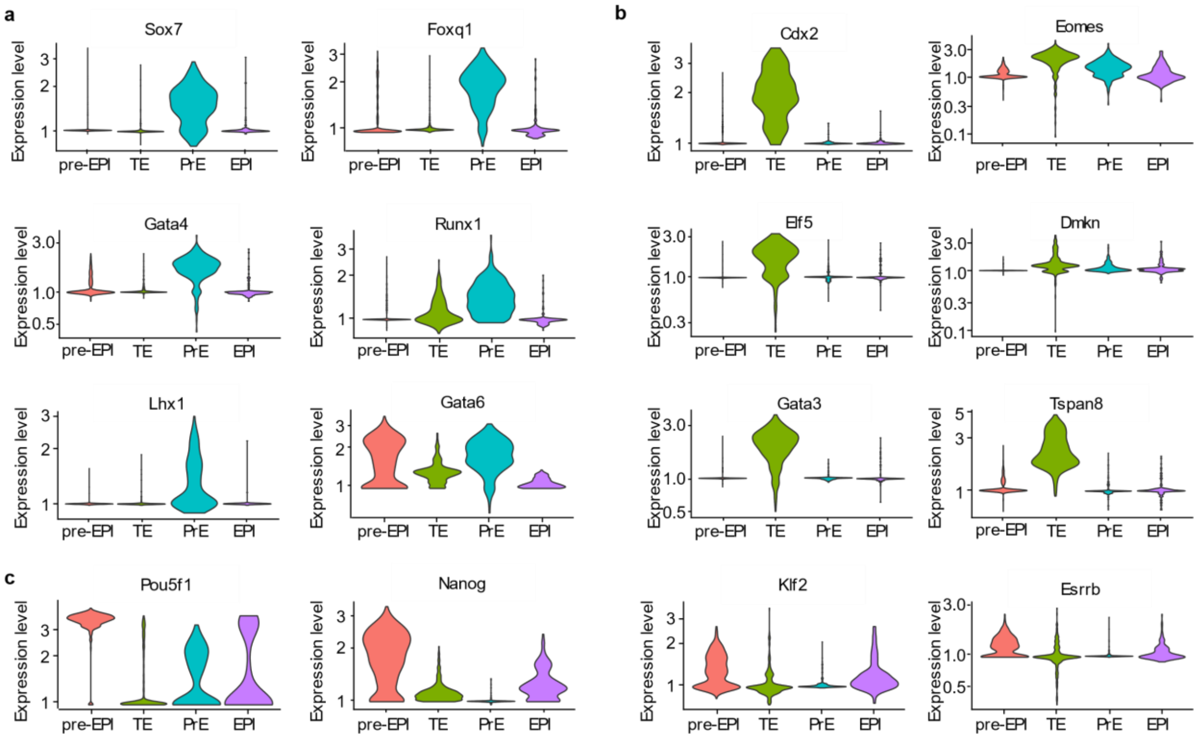

Figure S10. The identification of different cell types of IVC day 2 embryos on PDCO by specific marker genes. Violin plot showing the expression of each marker gene in different cell types, PrE (a, *Sox7*, *Foxq1*, *Lhx1*, *Gata4*, *Gata6*, and *Runx1*), TE (b, *Cdx2*, *Eomes*, *Elf5*, *Dmkn*, *Gata3*, *and Tspan8*), and pre-EPI and EPI (c, *Pou5f1*, *Nanog*, *Kllf2*, *and Esrrb*). All expression of the genes were determined by scRNA-seq with reference to E3.5-E4.5 embryos, and the cell number of each lineage type at distinct groups were detailed in Table S1. TE, trophectoderm; PrE, primitive endoderm; pre-EPI, epiblast at preimplantation stage; EPI, epiblast.

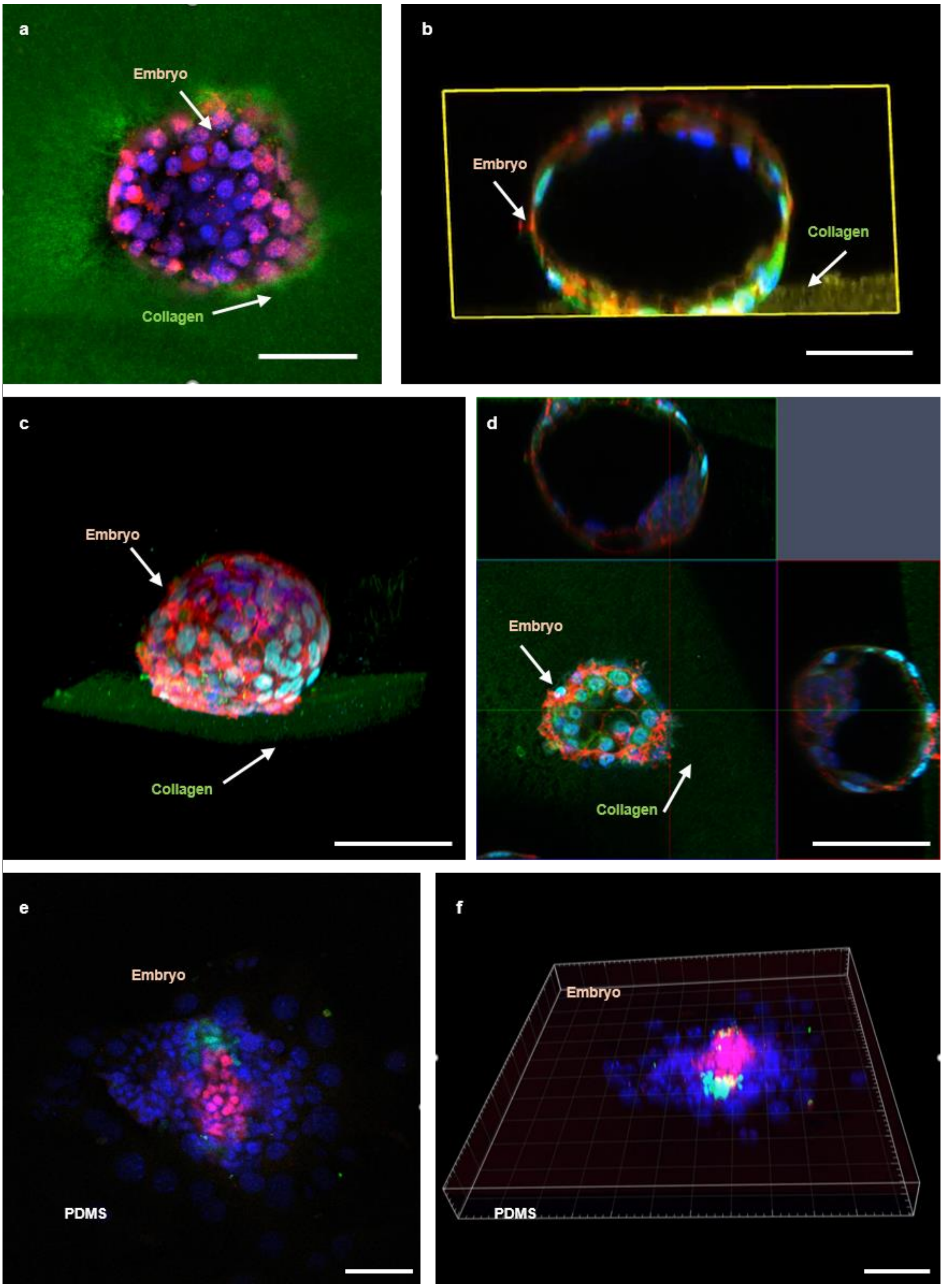

Figure S11. Representative fluorescence images of embryos invading into PDCO on IVC day 2. a,b) Screenshots of 3D confocal image movie (Movie S5) showing the embryonic invasion into collagen gel at the top view (a) and perpendicular view (b). The embryos are stained by antibodies of CDX2 (red, trophoblast) and Collagen I (green top layer materials of PDCO), and DAPI (blue, nucleus). n = 3 embryos. Scale bars, 100 µm. c) 3D images of PDCO embryos on IVC day 2 by staining trophoblast marker CDX2 (cyan), cytoskeleton marker Phall (red), top

layer materials of PDCO Collagen I (green) with DAPI (blue) visualizing nuclei. d) Perpendicular views of 3D image in c. n = 5 embryos in (c) and (d). Scale bars, 100 μm. e,f) Representative fluorescence images of PD embryos on IVC day 2. A screenshot of Movie S6 showing excessive trophoblast growth at the top (e) and side (f) views. OCT4, green (epiblast); CDX2, red (trophoblast) and DAPI, blue (nucleus). n = 3 embryos. Scale bars, 100 μm.

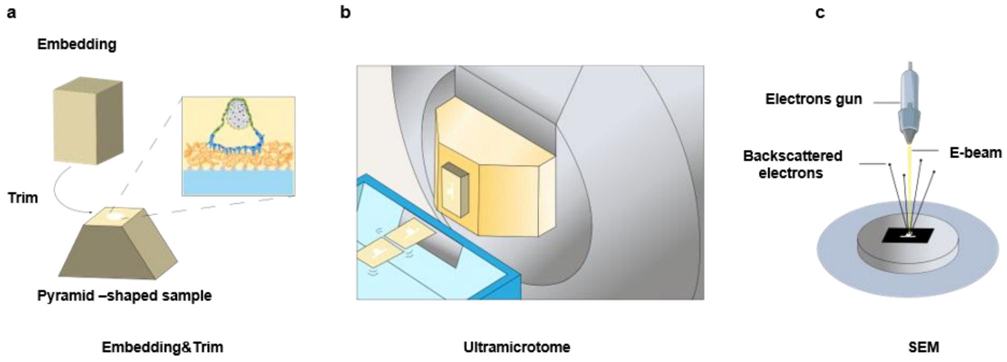

Figure S12. Schematic diagram of the sample preparation for sectional SEM. a) IVC embryos are infiltrated and embedded in the resin and trimmed to a suitable shape after curing. b) Samples are sectioned to 50-70 nm sections on thickness by ultramicrotome. **c,** Mounted sections imaged by FE-SEM under automated control after labeling and coating.

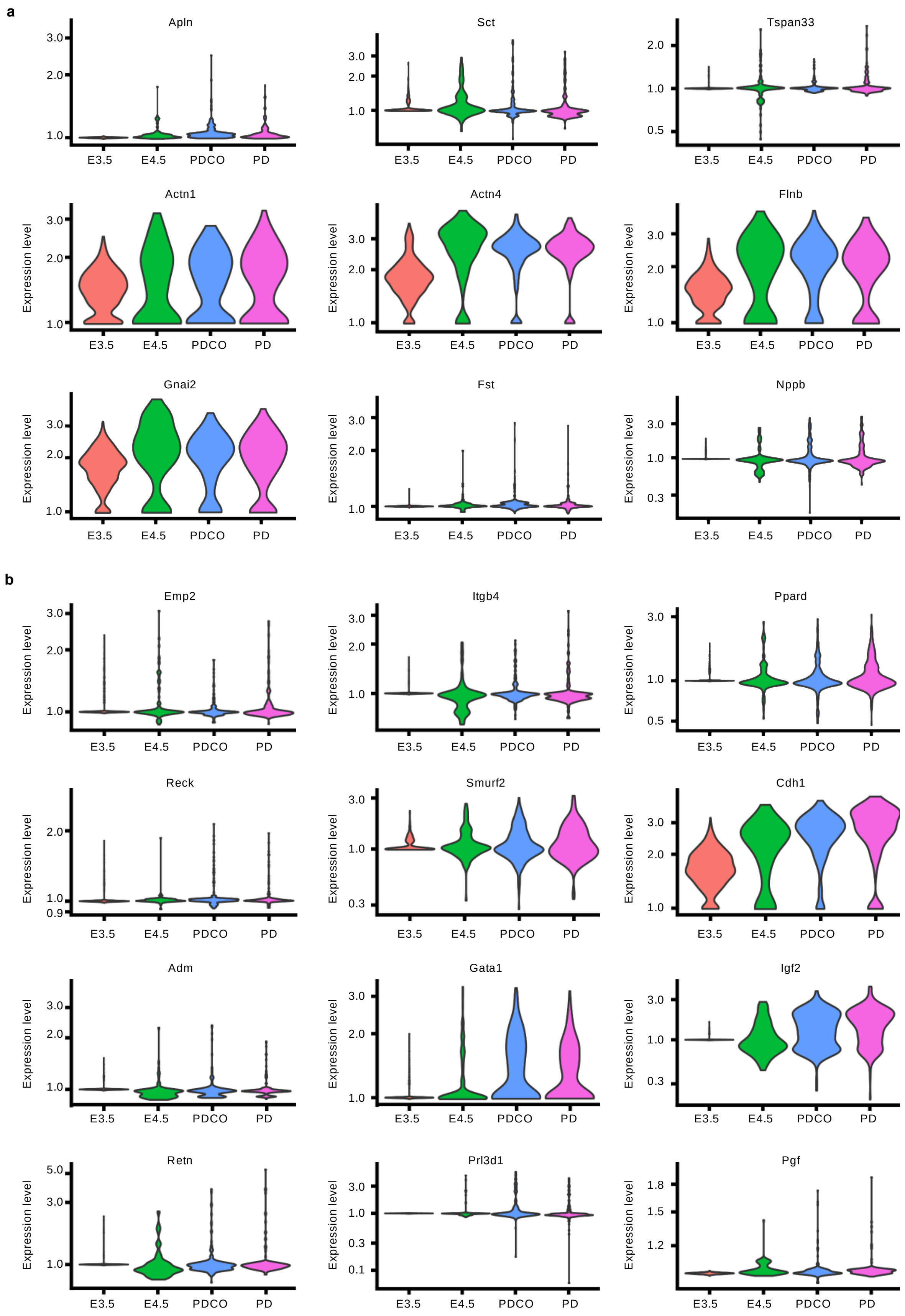
a
Apln
Sct
Tspan33
Actn1
Actn4
Flnb
Gnai2
Fst
Nppb
b
Emp2
Itgb4
Ppard
Reck
Smurf2
Cdh1
Adm
Gata1
Igf2
Retn
Prl3d1
Pgf
Expression level
E3.5
E4.5
PDCO
PD

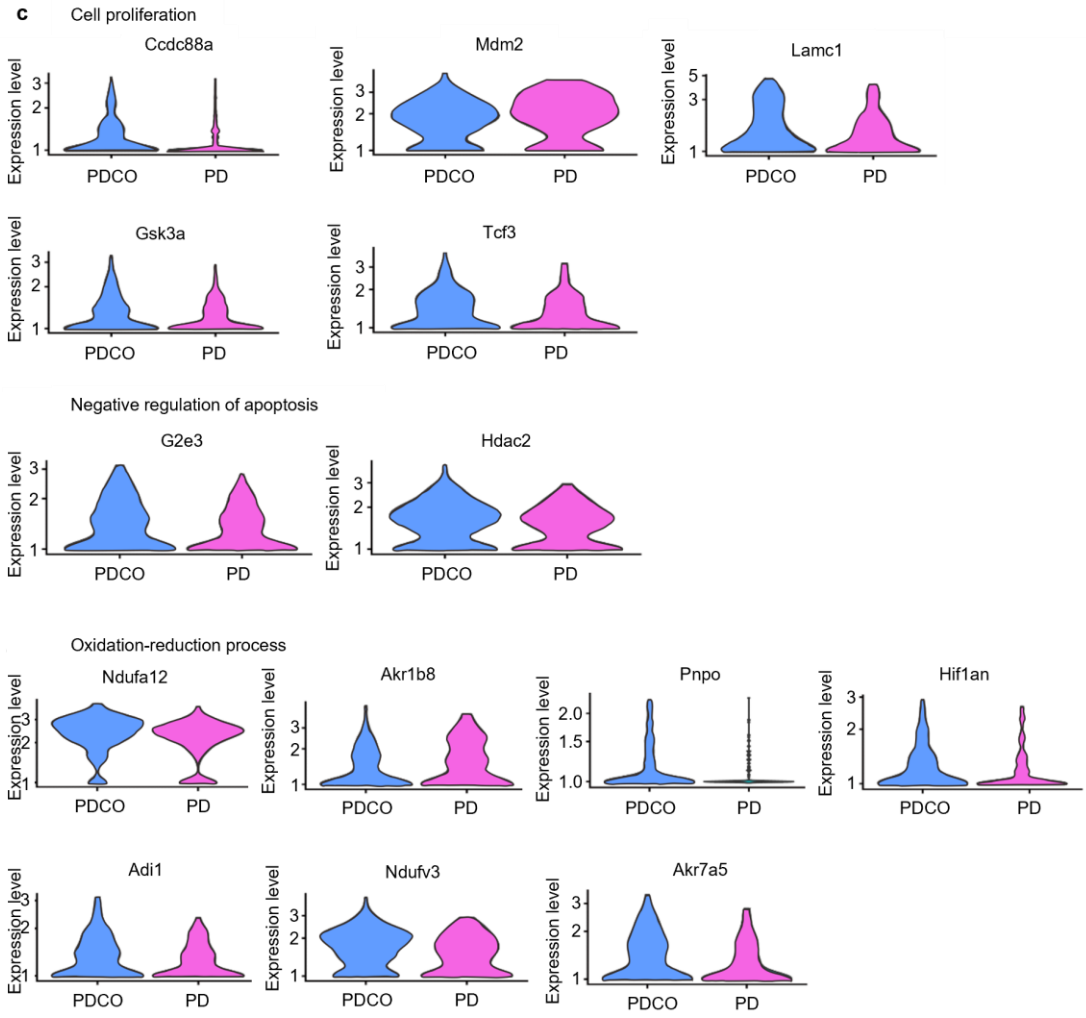

Figure S13. Gene expression related to hormone secretion and implantation in indicated embryos. a,b) Violin plots detailing the expression levels of hormone-related genes (a, *Apln, Sct, Tspan33*, *Actn1*, *Actn4*, *Finb*, *Gnai2*, *Fst*, and *Nppb*) and implantation-associated genes (b, *Emp2*, *Itgb4*, *Cdh1*, *Igf2*, *Ppard*, *Reck*, *Smurf2*, *Adm*, *Gata1*, *Retn*, *Prl3d1* and *Pgf*) in PD or PDCO embryos on IVC day 2, with reference to E3.5 and E4.5 embryos. c) Violin maps showing the expression of DEGs that regulating the “cell proliferation”, “negative regulation of apoptosis”, and “oxidation-reduction process” in single cells in PDCO and PD embryos.

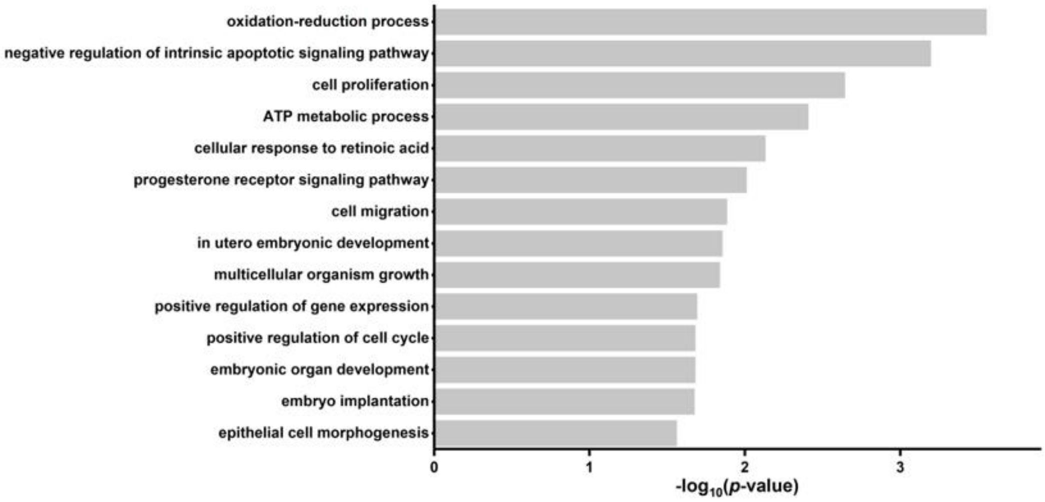

Figure S14. GO analysis of differentially up-expressed genes in PDCO embryos compared with PD ones on IVC day 2.

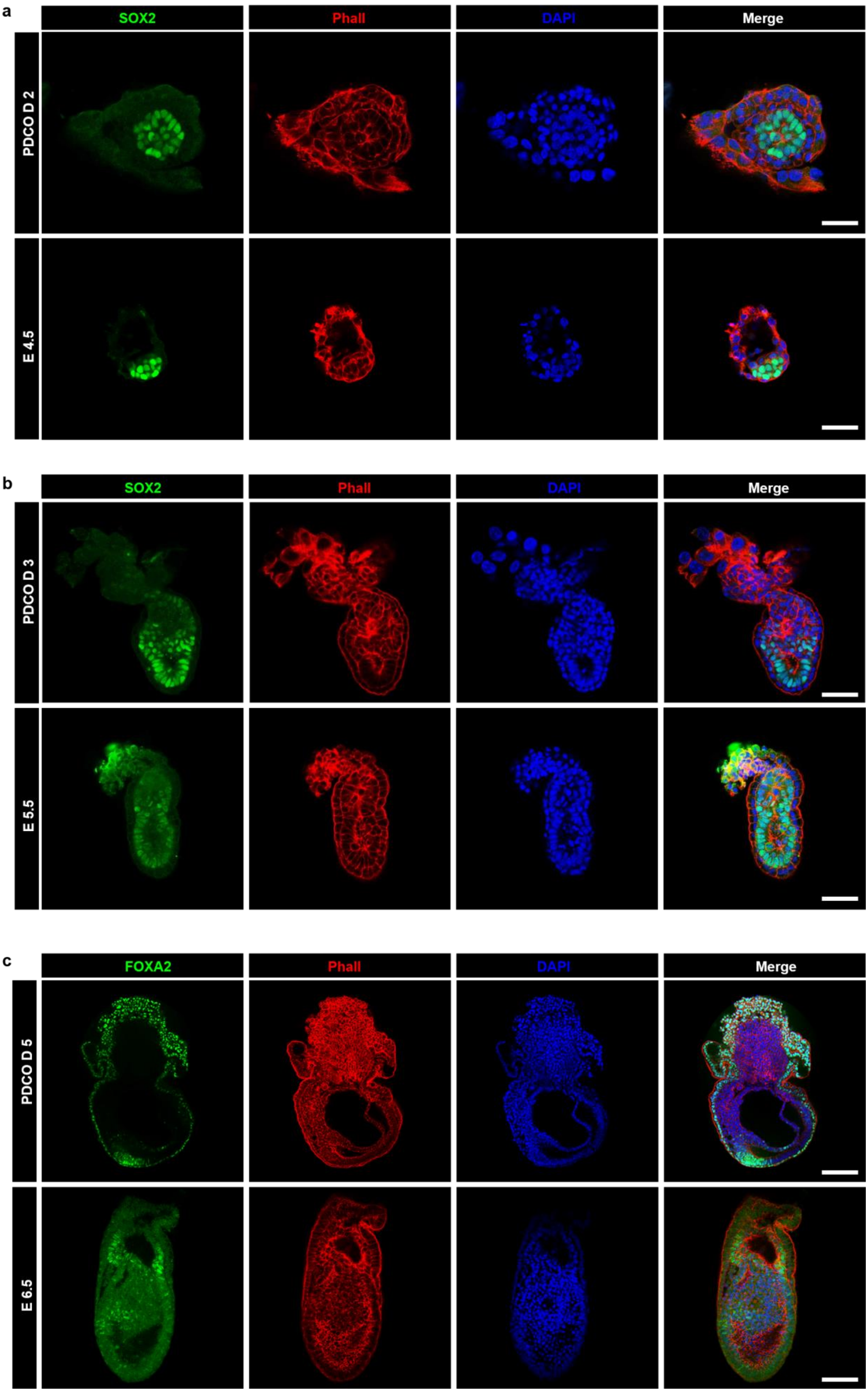
a
SOX2
Phall
DAPI
Merge
PDCO D 2
E 4.5
b
SOX2
Phall
DAPI
Merge
PDCO D 3
E 5.5
c
FOXA2
Phall
DAPI
Merge
PDCO D 5
E 6.5

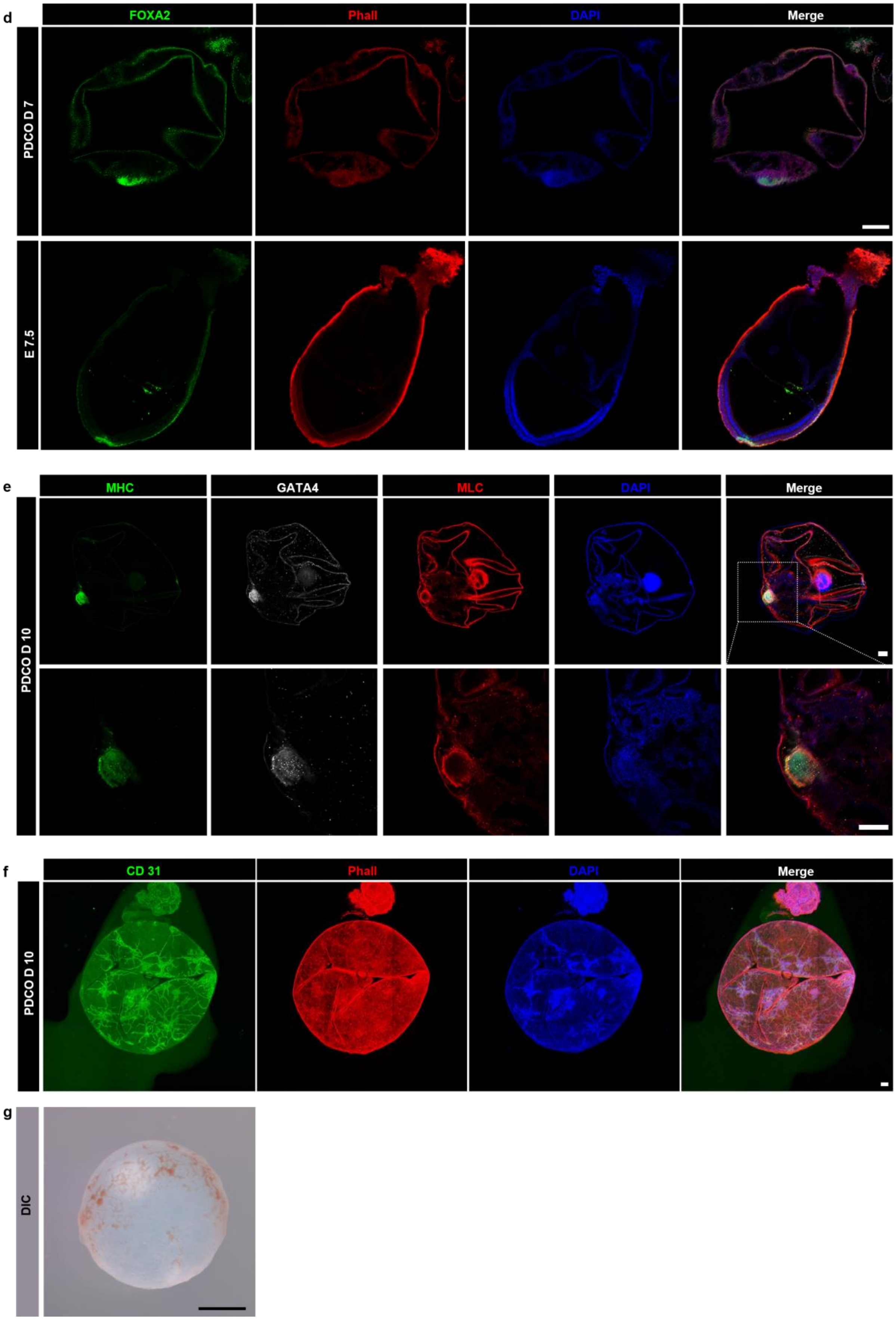
d
FOXA2
Phall
DAPI
Merge
PDCO D 7
E 7.5
e
MHC
GATA4
MLC
DAPI
Merge
PDCO D 10
f
CD 31
Phall
DAPI
Merge
PDCO D 10
g
DIC

Figure S15. Immunostaining IVC embryos on PDCO from the postimplantation to early organogenesis stage. a,b) IVC day 2 and day 3 embryos on PDCO and *in vivo* E4.5 and E5.5 embryos labeled by antibodies of SOX2 (green, labeling trophoblast) and Phall (red, labeling cytoskeleton) with nucleus visualization by DAPI (blue). n = 15 embryos per condition. Scale bars, 50 µm. c,d) IVC day 5 and day 7 embryos on PDCO and *in vivo* E6.5 and E7.5 embryos are stained by antibodies of FOXA2 (green, labeling primitive streak cells), and Phall (red). n = 10 embryos per condition. Scale bars, 50 µm. e) PDCO embryos on IVC day 10 are stained by antibodies of cardiomyocyte specific markers including MHC (green), GATA4 (gray), and MLC (red), compared to *in vivo* E8.5 embryos. Dotted line boxes, magnified region indicating the heart tube like structure. n = 4 embryos per condition. Scale bars, 100 µm. f) PDCO embryos are immunostained by endothelial cell marker CD31 (green) and cytoskeleton Phall (red) with DAPI (blue) visualizing nuclei on IVC day 10. n = 6 embryos. Scale bars, 100 µm. g) Bright-field image of a PDCO embryo on IVC day 8 showing red hematopoietic cells in the yolk sac. n = 8 embryos. Scale bar, 1 mm.

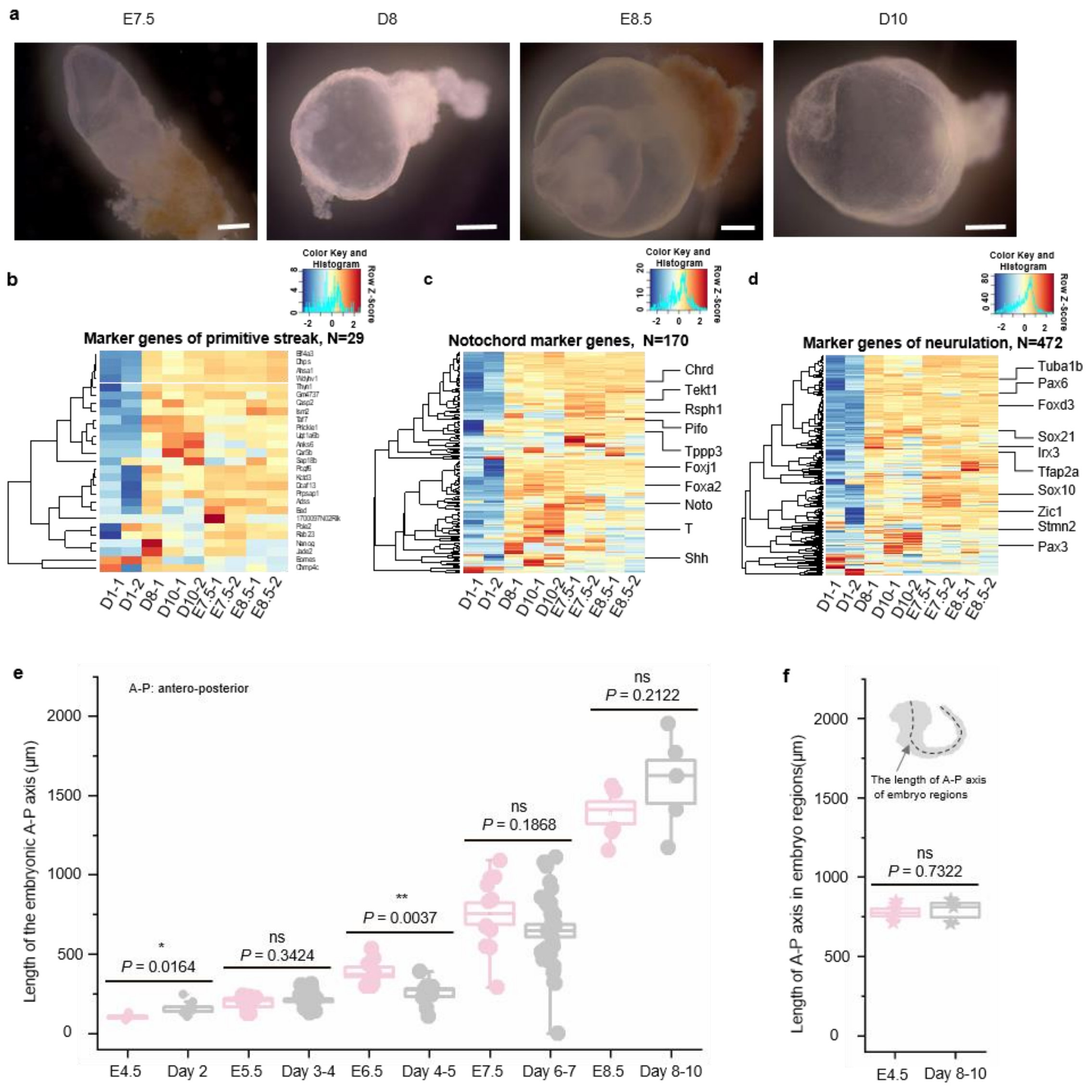

Figure S16. Bulk RNA-sequencing of mouse embryos on IVC days 8-10 and *in vivo* embryos at E7.5-8.5. a, Bight-field images of embryos collected for bulk RNA-sequencing. **b-d**, Heat maps showing the expression of marker genes for primitive steak (**b**), notochord (**c**), and neurulation (**d**) in PDCO embryos on IVC day 8 (D8) and day 10 (D10) and *in vivo* E8.5 embryos. D10-1,-2 for PDCO embryos on IVC day 10; E7.5-1,-2 for *in vivo* embryos at E7.5 and E8.5-1,-2 for *in vivo* embryos at E8.5. D1-1,-2 served as the negative control for the embryos at advanced developmental stages. **e**,**f**, The size of embryos (**e**) and embryo regions (**f**) *in vivo* and *in vitro* at the indicated time points. Diagrams depicting the A-P axis of embryos (Fig. 6**a**) and embryo regions (**f**) measured at corresponding stages. A-P: antero-posterior. *In vivo* embryos, n = 6 (E4.5), 7 (E5.5), 8 (E6.5), 12 (E7.5), 6 (E8.5) (left to right); IVC embryos,

n = 9 (Day 2), 30 (Day 3-4), 10 (Day 4-5), 35 (Day 6-7), 5 (Day 8-10); One-way ANOVA with Bonferroni post hoc test. ns, no significance; ns, $P > 0.05$; *$P < 0.05$; **$P < 0.01$.

Table S1

The cell number of each cell lineage at distinct groups after filtering in scRNA-seq analyses.

Sample	Pre-EPI	EPI	TE	PrE	Total
PD	5	30	99	49	183
PDCO	3	65	85	67	220
E4.5	7	26	56	26	115
E3.5_ref	696	2	76	0	774
E4.5_ref	1	49	28	154	232

Table S2

GO terms of genes upregulated in PDCO embryos on IVC day 2 compared with PD ones.

**Terms**	**Genes**
Oxidation-reduction process	*ALDH18A1, NDUFB7, PYROXD1, CHCHD4, FTH1, NDUFB2, UQCR11, HIF1AN, HMOX1, PNPO, AKR7A5, CAT, ETFB, GPD2, NDUFA4, SEPW1, GDI2, PTGR1, NDUFB10, AIFM1, NDUFA7, NDUFC1, SOD1, TET1, NDUFA1, NDUFA12, NDUFA11, AKR1B8, ADI1, NDUFV3, TXNDC12, PYCR2, KDM2A, RRM2, JMJD1C*
Negative regulation of intrinsic apoptotic signaling pathway	*G2E3, HDAC2, PARL, NDUFA13, HELLS, NOC2L*
Cell proliferation	*MORF4L1, PTGES3, USPL1, MKI67, MAP2K1, NASP, HLCS, GRHL2, TACC2, LARP1, PURA, SRRT, DKC1, GNG2, H3F3B*
ATP metabolic process	*ATP5J2, ATP5O, ATP5G1, HSPA8, ATP5K, ATP5J*
Cellular response to retinoic acid	*WNT7B, HDAC2, HTRA2, NDUFA13, SNRNP70, ABL2, PHC1*
Progesterone receptor signaling pathway	*NEDD4, PHB, UBR5*
Cell migration	*CORO1B, CCDC88A, S1PR1, GSK3A, EFNB2, LAMC1, ARPC5, CD63, MDK, PTEN, ABL2, LCP1*
Embryonic development in utero	*FGFR2, MYO1E, TRIM28, GJB3, CDH1, PRKCSH, RBBP6, GRHL2, GPI1, YBX1, WNT7B, SALL4, KDM2A, ANKRD11, MEG3, SYF2*
Multicellular organism growth	*FGFR2, GPD2, KDM2A, KMT2C, ANKRD11, MEG3, H3F3B, RBBP6, GRHL2*
Positive regulation of gene expression	*FGFR2, PTGES3, HMGB2, MAP2K1, PHB, LEF1, CALR, HNRNPU, RPS3, DROSHA, WNT7B, ID2, KDM2A, UBR5, MDM2, HSPA4, TCF3, DNMT3B, HSPA8*
Embryonic organ development	*FGFR2, WNT7B, SYF2, RBBP6, GRHL2*
Positive regulation of cell cycle	*FGFR2, ID2, MDM2, CALR, TCF3*
Embryo implantation	*GRN, FKBP4, TRIM28, CDH1, H3F3B, SOD1*
Epithelial cell morphogenesis	*CDH1, JMJD1C, GRHL2, IFT88*

Table S3

KEGG of genes upregulated in PDCO embryos on IVC day 2 compared with PD ones.

**Terms**	**Genes**
Ribosome	*RPL17, RPL19, MRPS14, RPL14, RPL13, RPLP2, RPL37, RPL38, RPS3, RPS25, MRPL11, RPS27, RPS29, RPL8, RPL3, FAU, RPL10A, RPL7A, MRPL33, RPS27A, RPL26, RPS5, RPS18, MRPS18C, RPS19, RPL41, RPL18A, MRPL27, RPL13A, RPS14, RPL21, RPS15, RPS12, RPS11*
Spliceosome	*BCAS2, EFTUD2, PPIL1, LSM7, DDX39B, SNRPD2, SF3A1, HNRNPU, HNRNPM, PPIH, SRSF9, SYF2, DHX16, SLU7, ACIN1, SNRNP70, SNRNP27, THOC2, PRPF38B, HSPA8, DDX42*
Oxidative phosphorylation	*NDUFA4, ATP5J2, NDUFB10, NDUFB7, NDUFA7, COX7C, COX4I1, NDUFA13, ATP5G1, NDUFC1, NDUFA1, NDUFA12, NDUFB2, NDUFA11, NDUFV3, UQCR11, ATP5O, ATP5K, ATP5J*
Alzheimer's disease	*NDUFA4, NDUFB10, NDUFB7, NDUFA7, COX7C, COX4I1, NDUFA13, ATP5G1, NDUFC1, NDUFA1, NDUFA12, NDUFB2, CAPN1, NDUFA11, NDUFV3, UQCR11, BACE1, CALM3, ATP5O, CALM1, ATP5J*
Huntington's disease	*NDUFA4, POLR2F, NDUFB10, NDUFB7, POLR2J, NDUFA7, COX7C, COX4I1, NDUFA13, ATP5G1, NDUFC1, REST, SOD1, NDUFA1, NDUFA12, NDUFB2, NDUFA11, NDUFV3, UQCR11, HDAC2, ATP5O, ATP5J*
Parkinson's disease	*NDUFA4, NDUFB10, NDUFB7, NDUFA7, COX7C, COX4I1, NDUFA13, ATP5G1, NDUFC1, NDUFA1, NDUFA12, NDUFB2, NDUFA11, NDUFV3, UQCR11, HTRA2, ATP5O, ATP5J*
Proteasome	*PSMB6, PSME1, PSME2, PSMA4, PSMB3, PSMC1, PSMB2, POMP, PSME4*
Non-alcoholic fatty liver disease (NAFLD)	*NDUFA4, NDUFB10, NDUFB7, NDUFA7, COX7C, COX4I1, NDUFA13, NDUFC1, NDUFA1, NDUFA12, NDUFB2, NDUFA11, NDUFV3, UQCR11, GSK3A*
Protein processing in the endoplasmic reticulum	*HSP90AB1, GANAB, PDIA3, RRBP1, UBE4B, PDIA4, CALR, PRKCSH, SSR1, RBX1, CAPN1, HSP90B1, ATF6B, DNAJA1, HSPA8*
RNA transport	*EEF1A1, XPO1, EIF2S3Y, DDX39B, EIF5B, RANGAP1, EIF4G1, EIF3A, EIF4EBP1, UPF3B, RAE1, EIF1, PABPC1, ACIN1, THOC2*
Prostate cancer	*HSP90AB1, FGFR2, CCNE2, HSP90B1, MAP2K1, PDGFA, MDM2, LEF1, PTEN*
Glioma	*MAP2K1, PDGFA, CALM3, MDM2, CDK4, PTEN, CALM1*
mRNA surveillance pathway	*PPP1CA, UPF3B, GSPT1, DDX39B, PABPC1, ACIN1, PPP2R2D, CPSF1*

**Terms**	**Genes**
Bladder cancer	*MAP2K1, RASSF1, MDM2, CDH1, CDK4*
Estrogen signaling pathway	*HSP90AB1, HSP90B1, MAP2K1, FKBP4, ATF6B, CALM3, HSPA8, CALM1*
Ubiquitin mediated proteolysis	*NEDD4, UBE3A, UBE2K, UBR5, UBE4B, UBE2M, MDM2, SAE1, UBE3C, RBX1*
Antigen processing and presentation	*HSP90AB1, PSME1, PDIA3, PSME2, HSPA4, CALR, HSPA8*
Ribosome biogenesis in eukaryotes	*DROSHA, NOL6, XPO1, DKC1, NOP58, GNL3L, NOP56*
Hippo signaling pathway	*AJUBA, ACTB, WNT7B, PPP1CA, FRMD6, ID2, RASSF1, LEF1, CDH1, PPP2R2D*
p53 signaling pathway	*CCNE2, RRM2, MDM2, CDK4, PERP, PTEN*
RNA polymerase	*POLR2F, POLR3K, POLR2J, POLR3D*
Melanoma	*MAP2K1, PDGFA, MDM2, CDH1, CDK4, PTEN*

Legends for Supplementary Movies

Movie S1

The movie of representative stress-strain curve for uniaxial tensile test of the uterus. During the tensile test, the strain gradually increased (left panel) when the uterine sample was slowly stretched upward (right panel). The points on the stress-strain curve referred to the motion when the sample was stretched from the tensile test loading cell.

Movie S2

The movie shows zooming into the cross sections and lumen surfaces of the decellularized uterus (left panel) and the collagen at a 7.5 mg/mL (right panel) from the macro to the nanostructures by SEM images at magnifications from 50 to 25600.

Movie S3

SEM images of the lumen inner surface of the natural uterus. **a**, Zooming into the endometrial surface of the natural uterus from the macro to the nanostructures by SEM images at magnifications from 50 to 25600. **b-d**, The movie continuously shows SEM images (**b**, Scale bar, 2 μm) from the magnification of image **c** (Scale bar, 5 μm), which was selected from image **d** (Scale bar, 300 μm) as indicated by the dotted blue line.

Movie S4

SEM images of the cross-section of the natural uterus. **a**, Zooming into the cross-section of the natural uterus from the macro to the nanostructures by SEM images at magnifications from 50 to 25600. **b-d**, The movie continuously shows SEM image (**b**, Scale bar, 2 μm) from the magnification of image **c** (Scale bar, 5 μm), which area was selected from image **d** (Scale bar, 300 μm) as indicated by the dotted blue line.

Movie S5

PDCO embryos were viewed using the confocal microscope on IVC day 2 to reconstruct 3D images by Imaris software with rotation view of total 87 sections compressed by Z-stack (**a**), a

gallery show of the total 77 optical sections of perpendicular view to the plane of PDCO surface by X-stack (**b**) and Y-stack (**d**), and gallery show of the total 77 optical sections by X, Y, Z-stacks (**c**). CDX2, red (trophoblast), and Collagen I, green (material), DAPI, blue (nucleus) in figs **a**, **c**, **d**, and CDX2, cyan (trophoblast), Collagen I, yellow (material), Phall, red (cytoskeleton), DAPI, blue (nucleus) in fig **b**. n = 3 embryos; Scale bars, 100 μm.

Movie S6

3D imaging of PD embryos on IVC day 2. Gallery show of the total 80 optical sections by Z-stack (**a**) and rotation view of all sections compressed by Z-stack (**b**). OCT4, green (epiblast), CDX2, red (trophoblast), and DAPI, blue (nucleus). n = 3 embryos; Scale bar, 100 μm.

Movie S7

One embryo with a heartbeat-like beating cultured on PDCO after approximately 8 days of *in vitro* culture and zooming into the beating site through magnification.

Supplementary references

[1] E. J. Jenkinson, I. B. Wilson, *Nature* **1970,** *228*, 776.
[2] T. C. Wu, Y. J. Wan, I. Damjanov, *J. Embryol. Exp. Morphol.* **1981,** *65*, 105.
[3] D. D. Carson, J. P. Tang, S. Gay, *Dev. Biol.* **1988,** *127*, 368.
[4] Y. C. Hsu, *Dev. Biol.* **1973,** *33*, 403.
[5] S. A. Morris, S. Grewal, F. Barrios, S. N. Patankar, B. Strauss, L. Buttery, M. Alexander, K. M. Shakesheff, M. Zernicka-Goetz, *Nat. Commun.* **2012,** *3*, 673.
[6] I. Bedzhov, C. Y. Leung, M. Bialecka, M. Zernicka-Goetz, *Nat. Protoc.* **2014,** *9*, 2732.
[7] G. Y. Huang, F. Li, X. Zhao, Y. F. Ma, Y. H. Li, M. Lin, G. R. Jin, T. J. Lu, G. M. Genin, F. Xu, *Chem Rev* **2017,** *117*, 12764.
[8] L. F. Xiang, Y. Yin, Y. Zheng, Y. P. Ma, Y. G. Li, Z. G. Zhao, J. Q. Guo, Z. Y. Ai, Y. Y. Niu, K. Duan, J. J. He, S. C. Ren, D. Wu, Y. Bai, Z. C. Shang, X. Dai, W. Z. Ji, T. Q. Li, *Nature* **2020,** *577*, 537.
[9] K. S. Kolahi, A. Donjacour, X. W. Liu, W. K. Lin, R. K. Simbulan, E. Bloise, E. Maltepe, P. Rinaudo, *PLoS One* **2012,** *7*, 10.
[10] A. J. Engler, S. Sen, H. L. Sweeney, D. E. Discher, *Cell* **2006,** *126*, 677.
[11] K. Saha, A. J. Keung, E. F. Irwin, Y. Li, L. Little, D. V. Schaffer, K. E. Healy, *Biophys. J.* **2008,** *95*, 4426.
[12] S. Meehan, A. S. Nain, *Biophys. J.* **2014,** *107*, 2604.
[13] K. Sheets, S. Wunsch, C. Ng, A. S. Nain, *Acta Biomater.* **2013,** *9*, 7169.
[14] S. E. Winograd-Katz, R. Fassler, B. Geiger, K. R. Legate, *Nat. Rev. Mol. Cell Biol.* **2014,** *15*, 273.
[15] M. Aragona, T. Panciera, A. Manfrin, S. Giulitti, F. Michielin, N. Elvassore, S. Dupont, S. Piccolo, *Cell* **2013,** *154*, 1047.
[16] C. F. Guimaraes, L. Gasperini, A. P. Marques, R. L. Reis, *Nat. Rev. Mater.* **2020,** *5*, 351.
[17] K. M. Yamada, M. Sixt, *Nat. Rev. Mol. Cell Bio.* **2019,** *20*, 738.
[18] A. Chronopoulos, S. D. Thorpe, E. Cortes, D. Lachowski, A. J. Rice, V. V. Mykuliak, T. Rog, D. A. Lee, V. P. Hytonen, A. E. D. Hernandez, *Nat. Mater.* **2020,** *19*, 669.
[19] P. Romani, I. Brian, G. Santinon, A. Pocaterra, M. Audano, S. Pedretti, S. Mathieu, M. Forcato, S. Bicciato, J. B. Manneville, N. Mitro, S. Dupont, *Nat. Cell Biol.* **2019,** *21*, 338.
[20] N. B. M. Rafiq, Y. Nishimura, S. V. Plotnikov, V. Thiagarajan, Z. Zhang, S. D. Shi, M. Natarajan, V. Viasnoff, P. Kanchanawong, G. E. Jones, A. D. Bershadsky, *Nat. Mater.* **2019,** *18*, 638.
[21] Y. Zheng, X. F. Xue, Y. E. Shao, S. C. Wang, S. N. Esfahani, Z. D. Li, J. M. Muncie, J. N. Lakins, V. M. Weaver, D. L. Gumucio, J. P. Fu, *Nature* **2019,** *573*, 421.
[22] S. P. Zhang, T. Z. Chen, N. X. Chen, D. F. Gao, B. B. Shi, S. B. Kong, R. C. West, Y. Yuan, M. L. Zhi, Q. Q. Wei, J. Z. Xiang, H. Y. Mu, L. Yue, X. H. Lei, X. P. Wang, L. Zhong, H. Liang, S. Y. Cao, J. C. I. Belmonte, H. B. Wang, J. Y. Han, *Nat. Commun.* **2019,** *10*, 496.
[23] A. Manfrin, Y. Tabata, E. R. Paquet, A. R. Vuaridel, F. R. Rivest, F. Naef, M. P. Lutolf, *Nat. Methods* **2019,** *16*, 640.
[24] R. Li, C. Zhong, Y. Yu, H. Liu, M. Sakurai, L. Yu, Z. Min, L. Shi, Y. Wei, Y. Takahashi, H.-K. Liao, J. Qiao, H. Deng, E. Nuñez-Delicado, C. Rodriguez Esteban, J. Wu, J. C. Izpisua Belmonte, *Cell* **2019,** *179*, 687.
[25] L. Xiang, Y. Yin, Y. Zheng, Y. Ma, Y. Li, Z. Zhao, J. Guo, Z. Ai, Y. Niu, K. Duan, J. He, S. Ren, D. Wu, Y. Bai, Z. Shang, X. Dai, W. Ji, T. Li, *Nature* **2020,** *577*, 537.
[26] J. Rossant, P. P. L. Tam, *Cell Stem Cell* **2017,** *20*, 18.
[27] S. A. Morris, *Open Biol* **2017,** *7*, 170003.
[28] J. Rossant, *Nature* **2016,** *533*, 182.

[29] S. Reardon, *Nature* **2016,** *533*, 15.
[30] S. Piccolo, S. Dupont, M. Cordenonsi, *Physiol. Rev.* **2014,** *94*, 1287.
[31] S. Dupont, *Exp. Cell Res.* **2016,** *343*, 42.
[32] G. Peng, S. Suo, G. Cui, F. Yu, R. Wang, J. Chen, S. Chen, Z. Liu, G. Chen, Y. Qian, P. P. L. Tam, J. J. Han, N. Jing, *Nature* **2019,** *572*, 528.
[33] K. Kono, D. A. A. Tamashiro, V. B. Alarcon, *Dev. Biol.* **2014,** *394*, 142.
[34] N. A. Perez-Gonzalez, N. D. Rochman, K. Yao, J. X. Tao, M. T. T. Le, S. Flanaryi, L. Sablichi, B. Toler, E. Crentsil, F. Takaesu, B. Lambrus, J. Huang, V. Fu, P. Chengappa, T. M. Jones, A. J. Holland, S. An, D. Wirtz, R. J. Petrie, K. L. Guan, S. X. Sun, *J. Cell Biol.* **2019,** *218*, 3472.
[35] D. M. Logsdon, A. F. Ermisch, R. Kile, W. B. Schoolcraft, R. L. Krisher, Y. Yuan, *J. Assist. Reprod. Genet.* **2020,** *37*, 747.
